# Waterlogging tolerance in pigeonpea: current understanding of physiological responses, genetic resources, and genomic prospects

**DOI:** 10.3389/fpls.2026.1923994

**Published:** 2026-09-28

**Authors:** Rafat Sultana, Kulbhushan Saxena, Kamal Krishna Pal, Rinku Dey, Zafar Imam, M. Isabel Vales, Rabiya Parveen, Sweta Sinha, Vijai Pal Bhadana, Anupama J. Hingane, Basavaraj P.S, M. Iqbal R. Khan, K. Sammi Reddy

**Affiliations:** 1 School of Drought Stress Management, ICAR-National Institute of Abiotic Stress Management, Pune, India; 2 International Crops Research Institute for the Semi-Arid Tropics, Hyderabad, India; 3 Department of Genetics and Plant Breeding, Bihar Agricultural University, Sabour, Bihar, India; 4 Department of Horticultural Sciences, Texas A&M University, College Station, TX, United States; 5 ICAR-Indian Institute of Agricultural Biotechnology, Ranchi, India; 6 International Atomic Energy Agency, Vienna International Centre, Vienna, Austria; 7 Department of Botany, Jamia Hamdard, New Delhi, India

**Keywords:** aerenchyma formation, anaerobic metabolism, antioxidant enzymes, pigeonpea waterlogging tolerance, reactive oxygen species (ROS), stress-responsive genes

## Abstract

Waterlogging is an escalating threat to pigeonpea (*Cajanus cajan* L. Millsp.) production in tropical and subtropical regions, where increasingly erratic monsoon rainfall and poorly drained Vertisols frequently expose crops to root-zone hypoxia during germination and early vegetative growth. Waterlogging in pigeonpea has historically been associated with substantial production losses in affected areas; however, the magnitude of these losses depends on stress duration, developmental stage, genotype, environmental conditions, and post-stress recovery. Oxygen deprivation disrupts root respiration, energy metabolism, and symbiotic nitrogen fixation, yet the mechanistic basis of tolerance remains poorly resolved. This review distinguishes experimentally supported physiological and anatomical responses from transcriptome-derived molecular hypotheses through a five-tier evidence-classification framework (Categories I–V). Experimental studies provide Category I evidence for traits associated with waterlogging tolerance, including cortical aerenchyma formation, hypertrophied lenticels, adventitious rooting, enhanced fermentative metabolism, and antioxidant enzyme activity in tolerant genotypes. Transcriptomic and genomic resources identify putative candidate gene families, but no candidate gene has been functionally validated in pigeonpea through gene editing, transgenic analysis, or expression quantitative trait locus (eQTL) mapping. Phenotypic screening has identified promising tolerant germplasm, including ICPL 20241, ICP 5028, and ICPH 2740, providing resources for breeding. We propose priorities for strengthening the evidence base: standardized multi-stage phenotyping, temporally resolved transcriptomics, QTL mapping, functional validation of candidate genes, and integrated multi-omics approaches. This evidence-based framework provides a roadmap for moving from observed tolerance mechanisms toward experimentally validated molecular targets and their deployment in climate-resilient pigeonpea breeding.

## 1 Introduction

Increasing climate variability has intensified the frequency of transient flooding and soil oxygen deficiency across rainfed agricultural systems, posing a major threat to crop productivity and food security (Bailey-Serres and Voesenek, 2008; Bailey-Serres et al., 2012; Fukao et al., 2019). Oxygen limitation disrupts root respiration, nutrient uptake, and energy metabolism, ultimately impairing plant growth and yield stability (Loreti et al., 2018). Grain legumes are particularly susceptible to hypoxic stress because of their dependence on active root metabolism and symbiotic nitrogen fixation (Msehli et al., 2016; Pucciariello et al., 2019).

Pigeonpea (*Cajanus cajan* L. Millsp.) is a climate-critical crop widely cultivated across the tropics and semi-arid regions. In India, which accounts for approximately 70–77% of global pigeonpea production (FAOSTAT, 2023), the crop is predominantly grown during the monsoon season on Vertisols with poor internal drainage. Erratic monsoon rainfall, combined with the poorly drained Vertisols characteristic of many pigeonpea-growing tracts, increasingly exposes the crop to intermittent waterlogging (Kumar et al., 2017; Basavaraj et al., 2023). This exposure is consistent with the general intensification of transient flooding described for rainfed cropping systems, although crop- and region-specific quantification for pigeonpea-growing areas remains limited (Ahmed et al., 2013).

Pigeonpea serves as a major source of dietary protein, essential amino acids, and income for smallholder farmers in India, Myanmar, eastern Africa, and parts of Southeast Asia. In addition to its nutritional importance, the crop contributes to soil fertility through biological nitrogen fixation and improves soil structure via its deep rooting system. However, despite its adaptation to drought-prone environments, pigeonpea remains highly vulnerable to excess soil moisture, particularly in poorly drained Vertisols receiving 600–1500 mm annual rainfall (Basavaraj et al., 2023). As a rainy-season crop, it is frequently exposed to transient waterlogging from germination through early vegetative growth, which can severely impair seedling establishment, biomass accumulation, and ultimately grain yield (Khare et al., 2002). The early developmental stages are particularly susceptible to oxygen deficiency, and even short periods of waterlogging can cause substantial stand loss and delayed crop establishment (Chauhan et al., 1997). Earlier literature has reported production reductions of approximately 25–30% associated with waterlogging in pigeonpea (Sultana, 2010; Choudhary et al., 2011; Tyagi et al., 2023). However, the original basis of this estimate is not accompanied by sufficient information on geographical coverage, assessment period, denominator, or calculation procedure to permit independent verification. We therefore treat the 25–30% value as a historical reported estimate rather than as a quantitatively validated contemporary estimate.

Waterlogging disrupts multiple physiological processes in pigeonpea, including root respiration, photosynthesis, and nutrient uptake, with severity depending on stress duration and developmental stage. Excess water restricts gaseous diffusion between soil and atmosphere, leading to rapid depletion of oxygen and accumulation of carbon dioxide in the rhizosphere (Sharma et al., 2021). These hypoxic conditions impair root respiration, suppress nodulation and symbiotic nitrogen fixation, and inhibit nutrient uptake, triggering widespread metabolic dysfunction. Legume-rhizobia symbiosis is particularly waterlogging-susceptible because both partners depend on aerobic respiration for energy supply, a vulnerability reviewed generally for legumes by Pucciariello et al. (2019) but not yet characterized specifically in pigeonpea.

Plants respond to hypoxia by developing various adaptive mechanisms, both morphological and physiological, such as the formation of aerenchyma, hypertrophied lenticels, adventitious rooting, stem swelling, and increased shoot elongation, all of which improve internal oxygen diffusion and maintain root function during hypoxia (Manik et al., 2019). The molecular mechanisms of tolerance include coordinated regulation of fermentative metabolism, antioxidant defense systems, ethylene signalling pathways, and hypoxia-responsive transcriptional regulators (Sasidharan and Voesenek, 2015; Hasanuzzaman et al., 2020). A central upstream factor of this transcriptional response is the oxygen-sensing mechanism mediated by the Arg/N-degron proteolytic pathway, which was discovered in *Arabidopsis* and relies on the oxygen-dependent stability of Group VII Ethylene Response Factors (ERF-VIIs) regulated by plant cysteine oxidases (Gibbs et al., 2011; Licausi et al., 2011). This pathway represents a critical hypothesis for understanding hypoxia responses in pigeonpea, but its components have not yet been experimentally tested in the species.

The availability of the genome sequence and transcriptome profiling of pigeonpea, along with the discovery of large sets of polymorphisms, has led to identification of gene families involved in anaerobic metabolism, homeostasis of reactive oxygen species (ROS), and remodelling of root structure (Tyagi et al., 2023). However, even with increasing physiological and genetic understanding, the mechanisms through which metabolic buffering, signalling cascades, and structural adaptation interact during waterlogging tolerance remain poorly resolved in pigeonpea.

While genomic resources are available for drought tolerance and disease resistance, research on waterlogging tolerance in pigeonpea has been mostly limited to physiological aspects and has not led to the functional validation of any candidate genes. Belliappa et al. (2024) recently reviewed physiological and anatomical evidence for waterlogging tolerance in pigeonpea. The present review differs in three ways: (i) it applies a consistent five-tier classification of evidence (Categories I–V) throughout the text, tables, and figures, distinguishing established mechanisms from those hypothesized from transcriptome data or interspecies comparisons; (ii) it explicitly links genomic and transcriptomic resources to physiological evidence; and (iii) it identifies evidence gaps as a prioritized research agenda rather than presenting a generic inventory of omics technologies.

Despite the importance of pigeonpea and the increasing threat posed by waterlogging, no systematic review has critically assessed the strength of evidence supporting proposed tolerance mechanisms, distinguished experimentally validated traits from putative pathways, or defined a translational research framework linking validated physiological and molecular evidence with practical breeding applications. Accordingly, this review addresses four key questions: (1) Which physiological and anatomical traits have been experimentally validated in pigeonpea? (2) Which donor genotypes are reproducibly tolerant across developmental stages and environments? (3) What molecular evidence is genuinely pigeonpea-specific, as opposed to inferred from other species? (4) Which knowledge gaps prevent the translation of genomic information into molecular breeding tools?

## 2 Review methodology

### 2.1 Literature search and evidence selection

This work was conducted as a narrative review using systematic search principles to identify and synthesize literature relevant to waterlogging tolerance in pigeonpea (*Cajanus cajan*) (**Figure 1**). The literature search was structured to provide broad coverage of physiological, biochemical, genetic, genomic, and molecular aspects of waterlogging and hypoxia responses, while the final synthesis was guided by relevance to the scope and objectives of the review.

**Figure 1:**
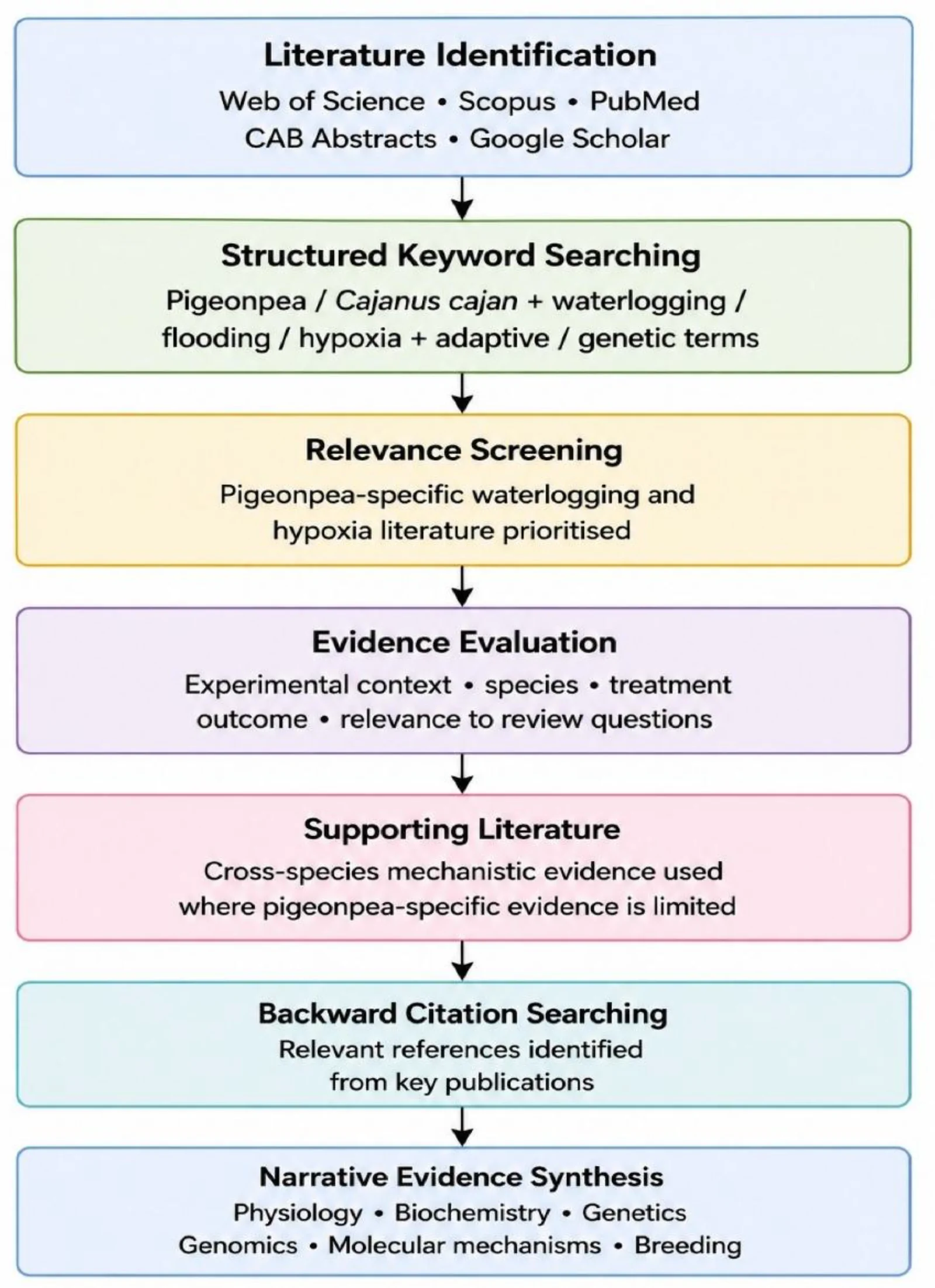
Literature identification, evaluation, and synthesis framework used in this narrative review conducted using systematic search principles.

Literature searches were conducted using Web of Science, Scopus, PubMed, CAB Abstracts, and Google Scholar. Boolean combinations of keywords related to the crop, stress condition, adaptive responses, and genetic mechanisms were used, including “pigeonpea”, “*Cajanus cajan*”, “waterlogging”, “flooding”, “hypoxia”, “anoxia”, “excess soil moisture”, “soil flooding”, “poor drainage”, “transient flooding”, “submergence tolerance”, “root-zone hypoxia”, “aerenchyma”, “adventitious root”, “germplasm screening”, and “genetic analysis”. The literature search was updated through 15 April 2026. No predefined publication-date restriction was applied.

Peer-reviewed original studies directly examining waterlogging, flooding, hypoxia, or associated adaptive responses in pigeonpea were prioritized. Pigeonpea genomic and transcriptomic resources were also considered when they provided information relevant to candidate genes, gene families, regulatory pathways, or other molecular components potentially associated with hypoxia adaptation. Studies of germplasm screening, physiological and biochemical responses, genetic variation, and breeding-related traits were included where they contributed directly to understanding waterlogging tolerance in pigeonpea.

Because experimentally validated molecular information on waterlogging tolerance in pigeonpea remains limited, selected studies from model plants and other crops, particularly *Arabidopsis*, rice, soybean, and other legumes, were used to provide mechanistic context. Such cross-species evidence is explicitly distinguished from pigeonpea-specific evidence and is not presented as direct experimental validation in pigeonpea. Review articles and methodological papers were used selectively to provide conceptual background and to identify relevant primary literature.

References were screened on the basis of their relevance to the major themes of the review, including morphological adaptation, physiological and biochemical responses, germplasm variation, genetic control, QTLs and genomic resources, transcriptional regulation, hypoxia-responsive metabolism, hormone and ROS signalling, and breeding applications. Reference lists of relevant publications were additionally examined to identify pertinent studies that might not have been retrieved during the initial literature searches.

Conference abstracts, theses, non-peer-reviewed reports, and sources lacking sufficient methodological or evidential information were excluded from the primary scientific evidence base. Where evidence from pigeonpea was unavailable, this limitation is explicitly acknowledged rather than inferred from evidence in other species. The literature was synthesized narratively rather than through a formal systematic-review or meta-analysis framework.

### 2.2 Evidence classification

All evidence was classified into five categories, applied uniformly throughout the text, tables, and figures: Category I: Experimentally observed or measured in pigeonpea under waterlogging (e.g., biochemical assays, anatomical observations, or physiological measurements in waterlogged pigeonpea plants, preferably with tolerant-versus-susceptible genotype comparisons); Category II: Differentially expressed or transcriptionally responsive in pigeonpea under waterlogging (e.g., RNA-seq or qRT-PCR data from waterlogging-treated pigeonpea, without independent functional validation); Category III: Identified in the pigeonpea genome or predicted from sequence homology, but not tested under waterlogging (e.g., gene-family annotations from genome papers); Category IV: Inferred from model plants or other crops (e.g., Arabidopsis, rice, soybean) and proposed as potentially relevant to pigeonpea based on orthology or conserved pathway logic and Category V: Proposed by the authors as a breeding hypothesis or conceptual framework, not yet experimentally tested.

This five-tier framework describes the origin and experimental status of evidence, not study quality or risk of bias. Studies within the same category may differ in experimental strength. Therefore, where information was available, study-level descriptors including replication, number of independent studies or environments, contrasting genotypes, developmental stage, environmental validation, recovery assessment, and yield measurement were considered separately. These descriptors provide additional context for the breadth and robustness of evidence without converting the evidence categories into a formal quality or risk-of-bias assessment.

### 2.3 Data analysis and figure preparation

All schematic figures, except **Figures 2**, **3**, were prepared in Microsoft PowerPoint using original schematic elements created by the authors. **Figure 2** was adapted from a previously published figure from a paper of co-author Hingane et al. (2015), whereas **Figure 3** was initially created using figure labs and subsequently edited and refined by the authors in Microsoft PowerPoint.

**Figure 2:**
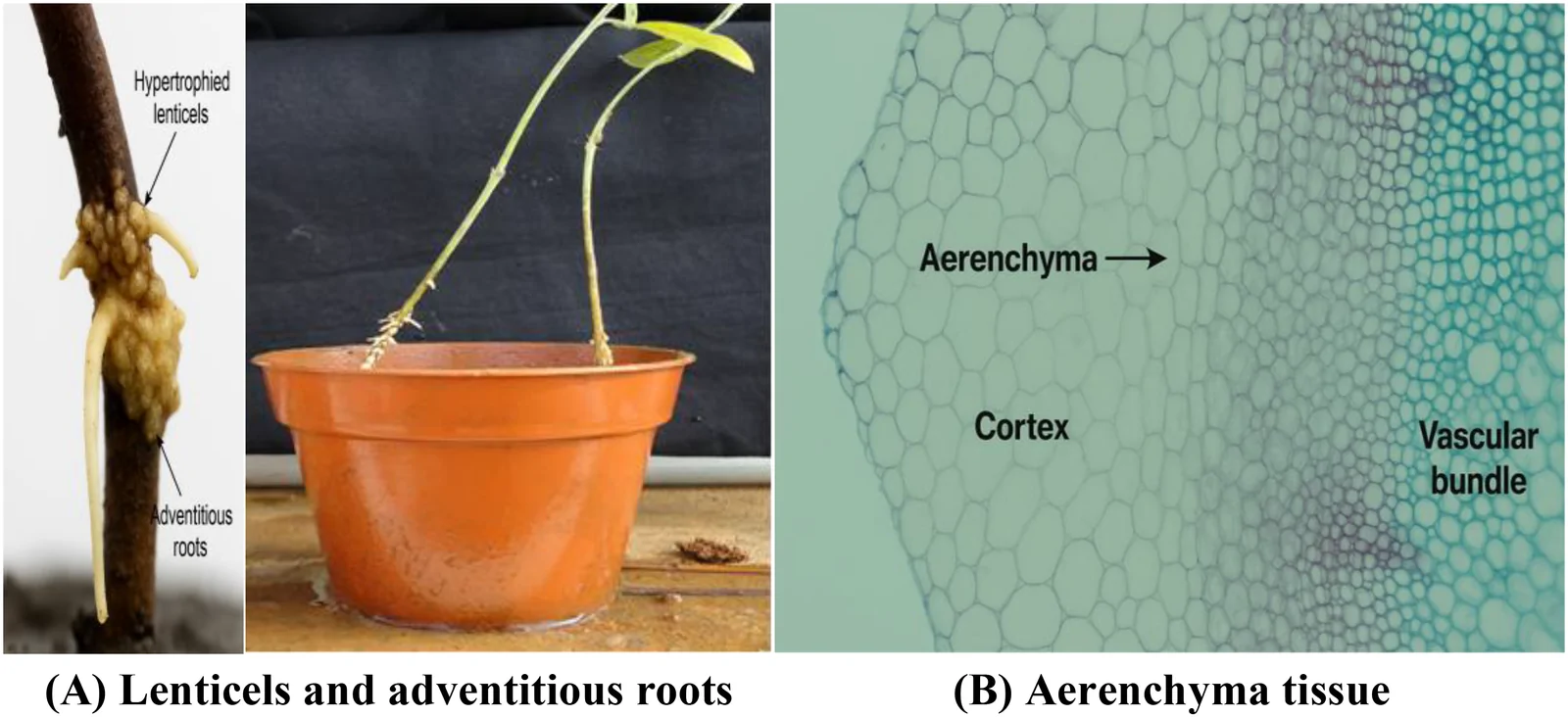
Anatomical adaptations to waterlogging in pigeonpea. **(A)** Schematic diagram of hypertrophied lenticels (arrowheads) and adventitious roots (arrows) on the stem base of genotype ICP 5028 after 14 days of pot waterlogging at the vegetative stage. **(B)** Schematic diagram of a transverse root section showing cortical aerenchyma (arrows) in genotype ICP 5028 after 10 days of waterlogging at the vegetative stage. This image is adapted from Hingane et al. (2015), not reproduced images. Scale bars = 1 mm **(A)** and 100 µm **(B)**. AR, adventitious root. Evidence: Category I (pigeonpea-specific observation).

**Figure 3:**
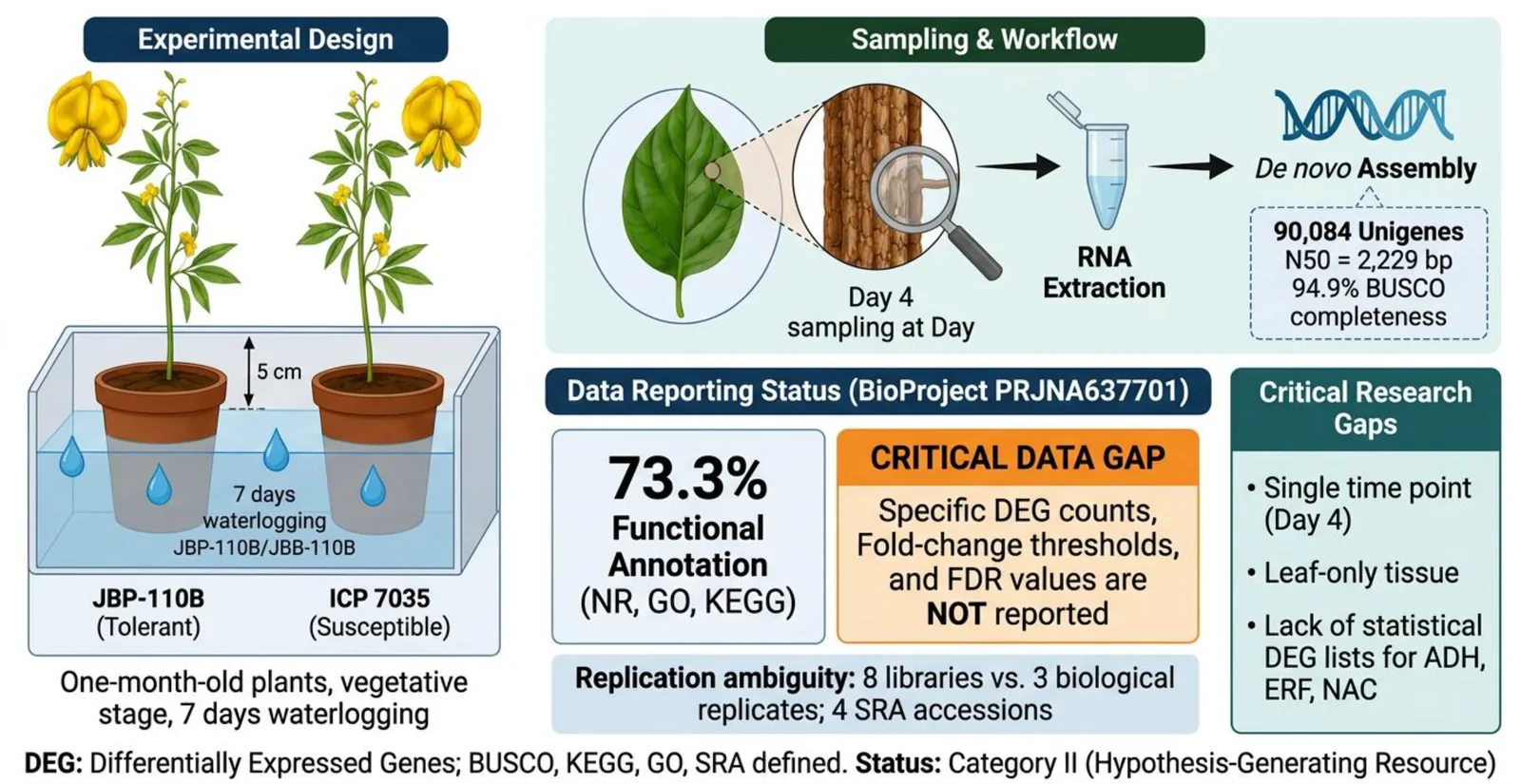
Schematic of the RNA-seq study design reported by Tyagi et al. (2023). Two contrasting genotypes, JBP-110B (tolerant) and ICP 7035 (susceptible), were subjected to 7 days of waterlogging at the vegetative stage (water level maintained at 5 cm above the soil surface). Leaf tissue was sampled at day 4 from control and waterlogged plants. The published Data Report describes *de novo* transcriptome assembly (90,084 unigenes; N50 = 2,229 bp), BUSCO completeness assessment, and GO/KEGG functional annotation of the assembled unigenes. The original article does not report specific differentially expressed gene counts, fold-change thresholds, false-discovery rates, lists of individual up- or down-regulated genes, or treatment-versus-control pathway enrichment statistics in its Results, tables, or figure legends.

## 3 Effects of waterlogging stress on pigeonpea growth and development

Waterlogging imposes severe constraints on pigeonpea growth primarily by creating hypoxic or anoxic conditions in the rhizosphere, as excess water restricts gaseous diffusion between soil and atmosphere. It is important to distinguish waterlogging (root-zone hypoxia in water-saturated soil) from complete submergence (shoots and roots under water), and hypoxia (low oxygen, typically <21% O_2_) from anoxia (absence of oxygen), because these conditions elicit different plant responses. The seed-submergence assay used in some screening studies does not reproduce root-zone waterlogging in an emerged seedling; tolerance at germination, vegetative growth, and flowering should therefore not be combined into a single generic phenotype.

Oxygen deprivation disrupts mitochondrial respiration and limits oxidative phosphorylation, leading to rapid depletion of cellular ATP reserves and metabolic imbalance (Kumutha et al., 2009; Tiwari et al., 2024). One of the earliest responses to hypoxia is impairment of root metabolism, manifested by reduced root elongation, browning of lateral roots, and progressive decay of the fine root system. In legumes, these effects are further exacerbated by the high oxygen demand of nitrogen-fixing nodules; consequently, waterlogging markedly suppresses nodulation efficiency and biological nitrogen fixation (Pucciariello et al., 2019). The major morphological, physiological, biochemical, and yield-related effects of waterlogging in pigeonpea are summarized in **Table 1**, with representative references indicating the evidence base for each category.

**Table 1:** Morphological, physiological, biochemical, and yield-related effects of waterlogging stress on pigeonpea (*Cajanus cajan*) across different growth stages.

Developmental stage	Waterlogging/submergence duration	Experimental system	Major response/outcome	Interpretation of evidence	Reference
Seed/germination	5–8 days (120–192 h)	*In vitro* seed submergence	Survival remained relatively high up to 5 days; substantial genotypic variation became evident with prolonged submergence, with differences among germplasm groups becoming more pronounced at 8 days	Direct evidence of duration-dependent variation at the earliest stage; 8-day submergence provides a stringent screening condition rather than a universal lethal threshold	Sultana et al., 2013
Seed/germination	2, 4, 6 and 8 days	*In vitro* seed submergence	Marked genotypic variation in survival; 33 of 162 genotypes survived 8 days and were subsequently advanced for pot screening	Direct evidence that tolerance can be selected across increasing durations at the seed stage	Basavaraj et al., 2023
Early vegetative (~20–25 DAS)	6 days	Pot waterlogging	Reduced photosynthesis, stomatal conductance, transpiration, chlorophyll and biochemical performance; tolerant genotype maintained better physiological status	Direct evidence of substantial physiological impairment after 6 days at early vegetative stage	Bansal and Srivastava, 2015
Early vegetative (~25 DAS)	2, 4 and 6 days + recovery	Pot waterlogging	Progressive changes in leaf area, dry matter, RWC, chlorophyll, membrane stability and oxidative stress; susceptible plants showed severe injury and poor recovery after prolonged stress	Strong evidence for a duration-dependent physiological and oxidative response; 4–6 days represent more severe stress than 2 days	Kumutha et al., 2009
Vegetative (~40 DAS)	6 days	Pot submergence/waterlogging	Strong genotype-dependent differences in mortality and root dry-mass reduction; susceptible ICP 7035 showed 63–100% mortality under different soil conditions, whereas ICP 8379 showed 0–38% mortality	Direct evidence that 6 days can be severe at the vegetative stage, but mortality was strongly genotype- and soil-dependent	Chauhan et al., 1997
Vegetative	Repeated 5- and 7-day cycles	Controlled partial submergence	Survival differed among genotypes after single and repeated stress cycles; ICAR PP-01, PP-02 and PP-03 maintained >50% survival after two 7-day cycles	Demonstrates that repeated transient waterlogging can impose greater cumulative selection pressure than a single exposure	Singh et al., 2022
Vegetative/physiological characterization	2, 4 and 6 days	Pot waterlogging	Progressive physiological, biochemical and oxidative changes with increasing duration	Supports a graded rather than binary response to duration	Kumutha et al., 2009; Sairam et al., 2009
Transient waterlogging/early crop growth	2–8 days	*In vitro* followed by pot screening	Increasing duration was used to identify genotypic variation and tolerant materials; selected genotypes were subsequently evaluated under pot conditions	Confirms that duration-specific screening can identify tolerant germplasm, but tolerance is genotype-dependent	Basavaraj et al., 2023
Field/crop establishment to later growth	Repeated 5–7-day partial submergence cycles	Controlled followed by waterlogged field conditions	Selected lines maintained survival and subsequently produced seed under field conditions	Provides evidence that tolerance to repeated short-term waterlogging can have agronomic relevance beyond survival alone	Singh et al., 2022

Waterlogging duration is not directly comparable among studies because stress intensity, water depth, soil type, genotype, plant age, and recovery period differed among experiments. Therefore, the durations presented here should be interpreted as experimentally tested exposure periods, rather than universal critical or lethal thresholds for pigeonpea.

Root dysfunction rapidly translates into whole-plant physiological disturbances. Under prolonged waterlogging, pigeonpea plants commonly exhibit leaf epinasty, premature wilting despite saturated soils, and chlorosis resulting from impaired chlorophyll synthesis and reduced nutrient acquisition. Stomatal closure and reduced mesophyll conductance limit carbon assimilation, while hormonal imbalance—particularly altered ethylene, abscisic acid, and auxin dynamics—accelerates senescence processes. Reproductive development is especially vulnerable, as hypoxia during flowering and pod formation leads to increased flower abscission, reduced pod set, and poor seed filling (Belliappa et al., 2024; Takele and McDavid, 1995). Consequently, yield losses of up to 30% have been reported under seedling-stage waterlogging in controlled experiments (Tyagi et al., 2023), depending on stress duration, severity, and crop developmental stage.

Beyond plant physiological responses, waterlogging triggers profound changes in soil biogeochemistry that further aggravate stress severity. Oxygen depletion shifts microbial metabolism toward anaerobic pathways, promoting denitrification and loss of available nitrogen as gaseous forms (Swathi et al., 2017). Reductive dissolution processes increase the solubility of iron (Fe^2+^) and manganese (Mn^2+^), often reaching phytotoxic concentrations, while sulfate-reducing bacteria generate hydrogen sulfide (H_2_S), which damages root tissues and inhibits nodulation (Cojean et al., 2020). Fluctuations in phosphorus availability and pH-dependent micronutrient dynamics further complicate nutrient acquisition. Thus, pigeonpea roots under waterlogging face a complex physiological paradox characterized by simultaneous nutrient deficiency, ionic toxicity, and impaired uptake capacity.

Post-drainage recovery remains largely uncharacterized in pigeonpea and represents an important knowledge gap. Reoxygenation after drainage causes a ROS burst, which can result in additional oxidative injury that outweighs the damage caused by hypoxia (León et al., 2021). There has been no systematic comparison of pigeonpea genotypes for recovery performance following drainage, creating a significant gap in understanding whether genotypes that survive floods can recover productivity.

The effects summarized in **Table 1** show significant differences across studies that hinder cross-study comparisons. Stress techniques differ greatly: some studies use pot-based waterlogging at the seedling stage (Singh et al., 2022; Basavaraj et al., 2023), while others use seed-submergence assays (Lal et al., 2018; Sultana et al., 2013), and field validation is rare. Available evidence indicates that the effect of waterlogging in pigeonpea is determined by an interaction between stress duration, developmental stage, genotype, and environmental conditions rather than by duration alone. At the seed stage, prolonged submergence of 5–8 days has been used to discriminate genotypic differences in survival, whereas at the early vegetative stage, 4–6 days of waterlogging produced substantial physiological and oxidative responses. At approximately 40 days after sowing, 6 days of waterlogging caused marked genotype-dependent differences in mortality and root injury, demonstrating that the same nominal duration can produce contrasting outcomes among genotypes and soil environments. Repeated 5–7-day partial-submergence cycles further indicate that cumulative stress may be more informative for screening than a single exposure.

This protocol heterogeneity likely contributes to varying yield-loss estimates. A direct comparison of rapeseed screening approaches revealed that seedling-establishment assays, seedling-stage pot tests, and field-yield evaluations capture different aspects of tolerance, with only limited association between early-stage survival and final output (Langan et al., 2022). Waterlogging during reproductive development often results in greater yield reduction than during vegetative development because earlier stress allows compensatory recovery mechanisms to function (Fukao et al., 2019). The contrast between waterlogging tolerance (survival, senescence delay) and post-drainage recovery capacity (ability to restore productivity after stress is removed) is especially important in pigeonpea but has yet to be characterized.

## 4 Anatomical and morphological adaptations enhancing internal aeration

Waterlogging imposes severe oxygen limitation on root systems, disrupting aerobic respiration, energy metabolism, and nutrient uptake. Survival under such hypoxic conditions depends on the ability of plants to rapidly deploy coordinated anatomical, metabolic, hormonal, and redox regulatory responses Based on the available evidence, tolerance in pigeonpea is proposed to depend on a combination of anatomical and metabolic adaptations that may involve early metabolic buffering followed by structural reorganization, a sequence hypothesized to restore internal oxygen diffusion and maintain root functionality (Category V).

### 4.1 Aerenchyma, lenticels, and adventitious roots

Formation of cortical aerenchyma is one of the most critical structural responses for plant adaptation to waterlogging, involving development of interconnected gas-filled spaces that facilitate longitudinal transport of oxygen from shoots to submerged roots and minimize resistance to diffusion (Ejiri et al., 2021; Yamauchi et al., 2019). In cereals and some legumes, lysigenous aerenchyma formation primarily arises from localized programmed cell death (PCD) within the cortex. In pigeonpea, aerenchyma has been observed in waterlogging-tolerant genotypes (e.g., ICPL 20241, ICP 5028) under waterlogging stress, but whether its formation involves lysigenous PCD, schizogenous separation, or both remains to be confirmed through histological and ultrastructural studies (Category I for observation; Category IV for mechanistic interpretation).

These gas-filled spaces increase root porosity while enhancing internal aeration under hypoxic conditions. Hypertrophied lenticels contribute an added benefit to the diffusion of oxygen into plant tissues. In model plants, ethylene-induced loosened cell walls and cortical expansion give rise to these larger openings, which preferentially occur just below the root-shoot junction (Jackson and Armstrong, 1999) (Category IV). Hypertrophied lenticels are reported in tolerant genotypes of pigeonpea under waterlogging stress (Hingane et al., 2015), supporting this as a pigeonpea-specific adaptation (Category I). Together, these structures improve internal oxygen diffusion during waterlogging. **Figures 2A, B** illustrates the principal anatomical adaptations observed in waterlogged pigeonpea.

Adventitious root formation represents another key anatomical adaptation. Under waterlogging stress, primary roots often deteriorate due to oxygen deprivation and accumulation of toxic metabolites. Adventitious roots emerge near the soil surface or above the flooded zone, where oxygen availability is comparatively higher. In model plants, ethylene-induced loosened cell walls and cortical expansion give rise to these larger openings, which preferentially occur just below the root-shoot junction (Jackson and Armstrong, 1999) (Category IV). Hypertrophied lenticels are reported in tolerant genotypes of pigeonpea under waterlogging stress (Hingane et al., 2015); the presence of hypertrophied lenticels is a pigeonpea-specific observation (Category I). Whether ethylene signalling mediates lenticel hypertrophy in pigeonpea, as hypothesized in model plants, remains untested.

Aerenchyma enhances porosity and oxygen diffusion, while adventitious roots replace primary roots that fail under hypoxia. Together, these traits form a complementary system that couples structural porosity with root renewal, associated with improved survival during extended flooding. Whether these traits are constitutive or inducible, their carbon costs, and their persistence after drainage remain unknown in pigeonpea and represent important research priorities.

The anatomical adaptations described above aerenchyma, lenticels, and adventitious roots represent complementary rather than redundant strategies, but their relative importance and potential trade-offs remain unresolved in pigeonpea. Aerenchyma provides a low-resistance internal oxygen pathway; lenticels serve as external gas-exchange interfaces; and adventitious roots replace oxygen-starved primary roots while themselves potentially containing aerenchyma. In cereals and some legumes, these traits are developmentally coordinated through ethylene signaling (Yamauchi and Nakazono, 2022), but in pigeonpea the temporal sequence whether lenticel hypertrophy precedes aerenchyma formation or vice versa and the genetic independence of these traits have not been examined). In cereals and some legumes, aerenchyma formation arises from lysigenous PCD (Category IV) and represents a loss of cortical tissue, while adventitious rooting diverts assimilates from shoot growth (Colmer and Voesenek, 2009). In pigeonpea, aerenchyma has been observed (Category I), but whether formation involves lysigenous PCD, schizogenous separation, or both remains unconfirmed. Notably, in kiwifruit (*Actinidia valvata*), tolerance was achieved through constitutively high fermentation enzyme activity and antioxidant capacity without aerenchyma formation, demonstrating that anatomical adaptations are not obligatory for waterlogging tolerance (Gao et al., 2023). Whether pigeonpea genotypes differ in their relative reliance on anatomical versus metabolic adaptations remains unknown and warrants experimental investigation.

## 5 Molecular and cellular mechanisms of waterlogging tolerance in pigeonpea

Waterlogging tolerance in pigeonpea depends on metabolic adjustments, redox regulation, hormone signaling, and transcriptional reprogramming that collectively sustain cellular function during oxygen deprivation. Under hypoxia, cells rapidly shift from aerobic respiration to glycolysis and fermentation to sustain ATP production. Concurrently, antioxidant enzymes are activated to scavenge reactive oxygen species generated during reoxygenation. Transcription factors coordinate these responses by regulating the expression of genes involved in fermentation, antioxidant defense, and programmed cell death. The relative contribution of each mechanism varies with stress duration, genotype, and developmental stage, and the specific genes and isoforms responsible remain largely unidentified in pigeonpea (**Figure 4**).

**Figure 4:**
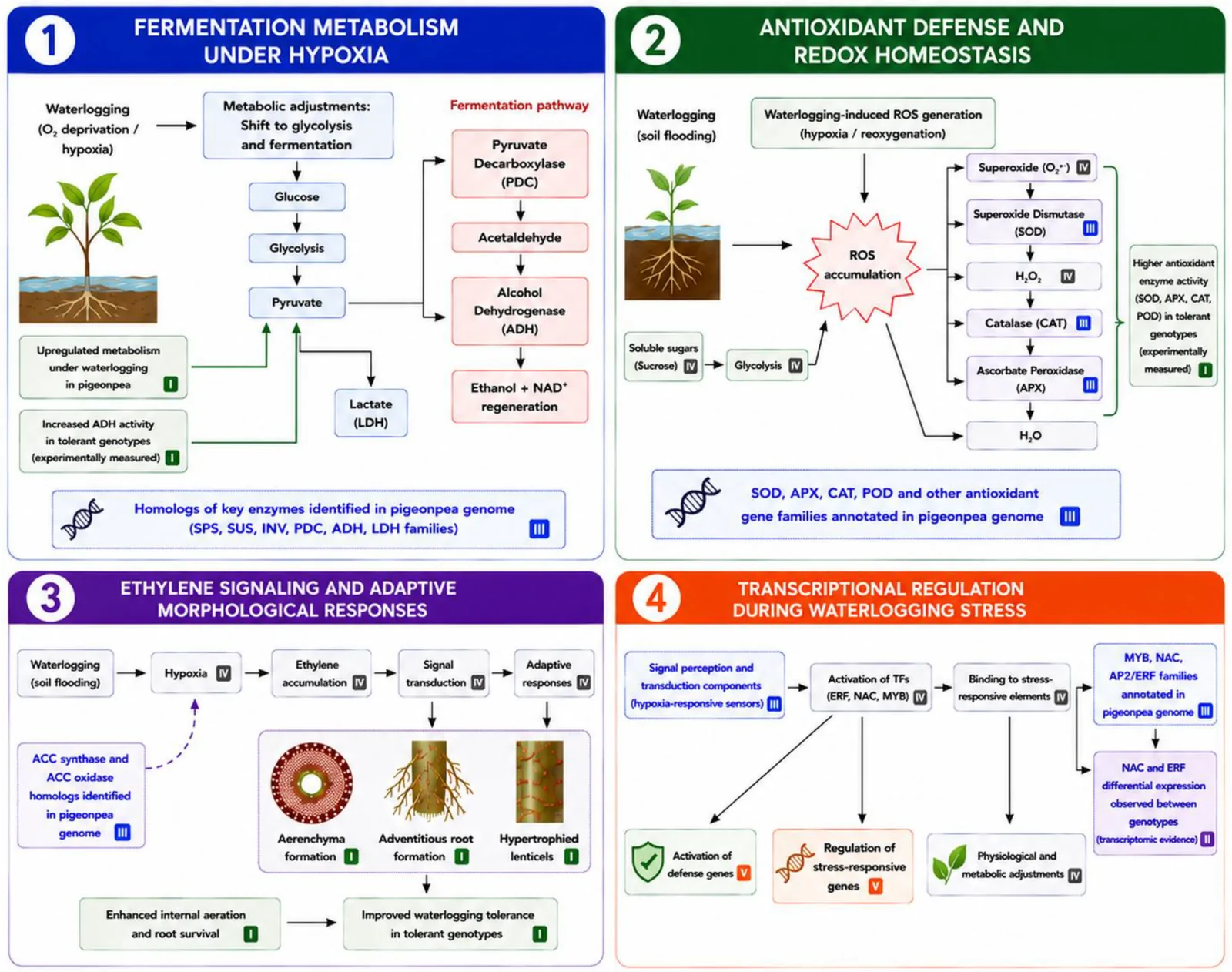
Major molecular and physiological pathways underlying waterlogging tolerance in pigeonpea (*Cajanus cajan*). Panel 1 illustrates the shift from aerobic metabolism toward glycolysis and fermentation under hypoxia, including PDC-, ADH-, and LDH-mediated pathways and experimentally observed metabolic responses. Panel 2 depicts waterlogging-induced ROS generation and antioxidant detoxification involving SOD, CAT, APX and related antioxidant systems. Panel 3 integrates hypoxia–ethylene signalling with aerenchyma formation, adventitious root development and hypertrophied lenticels as adaptive morphological responses. Panel 4 illustrates transcription factor regulation involving MYB, NAC and AP2/ERF families and their potential roles in stress-responsive and metabolic regulation. Evidence categories are assigned to individual claims or interactions rather than entire panels.

### 5.1 Carbohydrate and fermentation metabolism

Waterlogging-induced hypoxia restricts mitochondrial oxidative phosphorylation, forcing cells to shift from aerobic respiration to glycolysis and fermentative metabolism to sustain ATP production (**Figure 4**; Panel 1) (Pan et al., 2021) (Category IV). Soluble sugars derived from carbohydrate metabolism fuel glycolysis” is a simplification, while pyruvate is converted to acetaldehyde by pyruvate decarboxylase (PDC) and subsequently to ethanol by alcohol dehydrogenase (ADH), regenerating NAD^+^ to maintain glycolytic flux (Pan et al., 2021) (Category IV). Under hypoxia, starch and sucrose mobilization are also important, and this has not been studied in pigeonpea. Under acute oxygen deficiency, pyruvate may also be converted to lactate by lactate dehydrogenase (LDH) (Category IV). Although fermentation yields only ~2 ATP per glucose compared with ~30–32 ATP under aerobic respiration, it provides sufficient energy to maintain cellular viability during the early stages of flooding (Category IV).

Draft genome assemblies support the presence of putative homologs of key enzymes in these pathways (**Table 2**), although family-specific curation with traceable gene accessions is not yet available for most families. For carbohydrate metabolism, putative homologs related to sucrose phosphate synthase (SPS), sucrose synthase (SUS), and invertase (INV) have been inferred. Notably, Kumutha et al. (2008) provided Category I experimental evidence that SUS activity is upregulated in waterlogged pigeonpea, linking carbohydrate metabolism directly to the stress response. For fermentation, PDC, ADH, and LDH gene families encode the enzymatic machinery for pyruvate dissimilation. ADH enzyme activity is higher in waterlogging-tolerant pigeonpea genotypes than in susceptible lines (Bansal and Srivastava, 2015; Kumutha et al., 2008) (Category I), the specific ADH isoform(s) responsible for the increased activity observed in ICPL 84023 (tolerant genotypes) remain unknown, as the available assays measure total ADH activity without isoform resolution.

**Table 2:** Carbohydrate metabolism and fermentation pathways implicated in pigeonpea waterlogging responses.

Category	Pathway/enzyme	Biological function	Evidence in pigeonpea/(references)	Ortholog evidence (model species)/(references)
Carbohydrate metabolism	Sucrose phosphate synthase (SPS)	Catalyzes sucrose biosynthesis and regulates C allocation during stress	Potential homologs expected in the genome based on available draft assemblies (Varshney et al., 2011; Singh et al., 2012), but have not been systematically characterised in relation to waterlogging.	Not specifically implicated in hypoxia (At, Gm) (Bailey-Serres and Voesenek, 2008)
	Sucrose synthase (SUS)	Cleaves sucrose to supply substrates for glycolysis under O_2_ deprivation	Enzyme activity/protein level (Category I): Upregulated under waterlogging in pigeonpea (Kumutha et al., 2008). Gene identity (Category III): Genome assemblies predict carbohydrate metabolism genes (Singh et al., 2012), but responsible SUS isoforms have not been individually curated or functionally validated under waterlogging.	Hypoxia-inducible in Arabidopsis (Klok et al., 2002)
	Invertase (INV)	Hydrolyzes sucrose into glucose and fructose to sustain energy metabolism	Potential homologs expected in the genome based on available draft assemblies (Varshney et al., 2011; Singh et al., 2012), but have not been systematically characterised in relation to waterlogging.	Flooding-responsive in soybean (Komatsu et al., 2011)
Fermentation	Pyruvate decarboxylase (PDC)	Converts pyruvate to acetaldehyde during anaerobic fermentation	Potential homologs expected in the genome based on available draft assemblies (Varshney et al., 2011; Singh et al., 2012), but have not been systematically characterised in relation to waterlogging.	Core anaerobic protein in Arabidopsis, rice (Ismond et al., 2003; Quimio et al., 2000)
	Alcohol dehydrogenase (ADH)	Catalyzes ethanol formation and regenerates NAD^+^ under hypoxia	Enzyme activity (Category I): Increased total ADH activity reported under waterlogging (Bansal and Srivastava, 2015; Kumutha et al., 2008). Gene identity (Category III): Potential homologs expected in the genome based on available draft assemblies (Varshney et al., 2011; Singh et al., 2012), but responsible isoforms have not been systematically curated or functionally validated under waterlogging.	Essential for hypoxia tolerance in Arabidopsis, soybean, rice (Ismond et al., 2003; Fukao et al., 2019)
	Lactate dehydrogenase (LDH)	Converts pyruvate to lactate during early hypoxic stress	Potential homologs expected in the genome based on available draft assemblies (Varshney et al., 2011; Singh et al., 2012), but have not been systematically characterised in relation to waterlogging.	Early hypoxia response in Arabidopsis (Ismond et al., 2003)

Draft genome assemblies (Varshney et al., 2011; Singh et al., 2012) establish that genomic resources exist for pigeonpea, but they do not provide systematically curated, traceable inventories of individual waterlogging-responsive members for every family listed. Entries for SPS, INV, PDC, and LDH should therefore be regarded as putative homologs that have not been characterised in relation to waterlogging. SUS and ADH entries combine Category I (experimental enzyme/protein evidence in waterlogged pigeonpea) with Category III (unidentified isoforms). The Ortholog Evidence column indicates whether a hypoxia- or flooding-related function has been experimentally validated in model species (Category IV).

### 5.2 Antioxidant defense and redox homeostasis

Waterlogging-induced hypoxia and subsequent reoxygenation lead to excessive accumulation of reactive oxygen species (ROS), which can damage cellular membranes, proteins, and nucleic acids if not efficiently scavenged (Sairam et al., 2008; León et al., 2021). Draft genome assemblies indicate that pigeonpea possesses gene families related to antioxidant defense (**Table 3**; Panel 2), but systematically curated inventories with traceable accessions for waterlogging-responsive members are not yet available.

**Table 3:** Antioxidant enzyme families implicated in reactive oxygen species detoxification in pigeonpea under waterlogging stress.

Gene family	Role	Evidence in pigeonpea/(references)	Ortholog evidence (model species)/(references)
SOD	Superoxide dismutation	Enzyme activity (Category I): Significantly higher in tolerant than in susceptible waterlogged genotypes (Sairam et al., 2009). Gene identity (Category III): Responsible isoforms unidentified (Varshney et al., 2011; Singh et al., 2012).	Hypoxia/reoxygenation response in Arabidopsis, soybean, rice (León et al., 2021; Gill and Tuteja, 2010)
CAT	H_2_O_2_ decomposition	Enzyme activity (Category I): Significantly higher in tolerant than in susceptible waterlogged genotypes (Sairam et al., 2009). Gene identity (Category III): Responsible isoforms unidentified.	H_2_O_2_ detoxification under hypoxia in Arabidopsis, rice (León et al., 2021)
POD	H_2_O_2_ reduction (peroxidase)	Enzyme activity (Category I): Altered peroxidase activity reported under waterlogging (Bansal and Srivastava, 2015); significantly higher in tolerant than in susceptible genotypes (Sairam et al., 2009). Gene identity (Category III): Responsible isoforms unidentified.	Peroxidase-mediated ROS detoxification under hypoxia (Mittler, 2002)
APX	Ascorbate-mediated H_2_O_2_ reduction	Enzyme activity (Category I): Significantly higher in tolerant than in susceptible waterlogged genotypes (Sairam et al., 2009). Gene identity (Category III): Responsible isoforms unidentified; no waterlogging-specific transcript expression data reported.	Ascorbate-mediated ROS detoxification under hypoxia in Arabidopsis (Mittler, 2002; León et al., 2021)
GPx	Peroxide detoxification	Putative homologs inferred from draft genome assemblies; no waterlogging-specific enzyme activity or expression data available (Category III) (Varshney et al., 2011; Singh et al., 2012).	Oxidative stress response in Arabidopsis; limited hypoxia-specific validation (Mittler, 2002)
GST	Glutathione conjugation	Putative homologs inferred from draft genome assemblies; no waterlogging-specific enzyme activity or expression data available (Category III) (Varshney et al., 2011; Singh et al., 2012; Dixon et al., 2010).	Flooding and oxidative stress in soybean, Arabidopsis; large family expansion may reflect general stress adaptation (Dixon et al., 2010; Komatsu et al., 2011)

Evidence in pigeonpea is presented at two distinct levels: (i) enzyme activity measurements in waterlogged plants (Category I biochemical evidence), and (ii) gene family membership (Category III genome-prediction evidence). Comparative enzyme activities of SOD, CAT, POD, and APX were reported to be significantly higher in waterlogged tolerant than in susceptible pigeonpea genotypes (Sairam et al., 2009; Bansal and Srivastava, 2015), but the responsible genes or isoforms remain unidentified (Category III). GPX and GST entries represent putative homologs inferred from genome resources without waterlogging-specific enzyme activity or expression data in pigeonpea (Category III). Ortholog evidence from model species (*Arabidopsis*, rice, soybean) is classified as Category IV.

Consistent with this genetic capacity, Sairam et al. (2009) reported significantly higher activities of SOD, CAT, POD, and APX in the waterlogging-tolerant genotype ICPL 84023 than in susceptible genotypes, providing Category I biochemical evidence that enhanced antioxidant capacity contributes to tolerance. Bansal and Srivastava (2015) additionally reported altered peroxidase activity under waterlogging in pigeonpea. However, the specific genes or isoforms responsible for these elevated activities remain unidentified (Category III). For GPX and GST, neither comparative enzyme activity assays nor expression data under waterlogging have been reported in pigeonpea, leaving their stress-responsive isoforms entirely speculative.

### 5.3 Hormonal and ROS-mediated developmental signalling

In model plants, ethylene is a key regulator of developmental adaptation to waterlogging (Category IV). Flood-induced hypoxia restricts gas diffusion, leading to ethylene accumulation that promotes adaptive traits such as aerenchyma formation, adventitious root development, and hypertrophied lenticels, thereby improving internal oxygen transport and plant survival (Sasidharan and Voesenek, 2015) (Category IV). In pigeonpea, tolerant genotypes develop these anatomical adaptations under waterlogging, whereas susceptible genotypes show limited responses (Tiwari et al., 2024) (Category I), but whether ethylene accumulation is the causal signal driving these responses in pigeonpea has not been demonstrated.

Although ethylene is a major regulator of plant responses to waterlogging, flooding tolerance is likely mediated through interactions among multiple phytohormonal pathways. Abscisic acid (ABA) contributes to stomatal regulation and osmotic adjustment, whereas auxin is associated with adventitious root formation and developmental plasticity under hypoxic conditions. Gibberellins can influence shoot elongation and growth responses during flooding, while jasmonates and salicylic acid have been implicated in stress signaling and systemic acclimation in other plant species. However, these hormonal responses remain poorly characterized in pigeonpea, and their relative contributions, interactions with ethylene, and genotype- and stage-specific effects are largely unresolved. Thus, the involvement of these hormones in pigeonpea waterlogging tolerance should currently be regarded as a research hypothesis supported primarily by evidence from other species, rather than as an experimentally established mechanism. Targeted physiological, hormonal, transcriptomic, and functional studies in contrasting pigeonpea genotypes are needed to determine whether these pathways contribute to tolerance and recovery.

Ethylene signalling is closely linked with calcium- and ROS-mediated signalling (**Figure 4**; Panel 3). In model plants, hypoxia-induced calcium influx activates calcium-dependent protein kinases (CDPKs) and respiratory burst oxidase homologs (RBOHs), generating localized ROS that function as signaling molecules to regulate cortical programmed cell death and lysigenous aerenchyma formation (Suzuki et al., 2012; Yamauchi and Nakazono, 2022) (Category IV). Putative homologs of ACS, ACO, CDPK, and RBOH are inferred from draft genome assemblies, but none have been functionally characterized or profiled under waterlogging in pigeonpea, leaving their specific roles entirely speculative (**Table 4**).

**Table 4:** Ethylene–reactive oxygen species signalling-related gene families in the pigeonpea genome.

Category	Gene family	Role	Evidence in pigeonpea/(references)	Ortholog evidence (model species)/(references)
Ethylene biosynthesis	ACS	ACC synthesis	Putative homologs inferred from draft genome assemblies; no family-specific curation or waterlogging-specific expression/functional data available (Varshney et al., 2011; Singh et al., 2012).	Flooding-inducible ethylene production in soybean, rice (Pan et al., 2021; Sasidharan and Voesenek, 2015)
	ACO	ACC oxidation to ethylene		Hypoxia-responsive in soybean, Arabidopsis (Pan et al., 2021; Yamauchi and Nakazono, 2022)
Calcium signalling	CDPK	Ca^2+^ signal transduction		Hypoxia-induced Ca^2+^ signalling and aerenchyma regulation in Arabidopsis, rice (Yu et al., 2024; Li et al., 2025)
ROS production	RBOH	NADPH oxidase; ROS generation		Apoplastic ROS signalling during hypoxia in Arabidopsis (Yu et al., 2024; Yamauchi et al., 2017)

Draft genome assemblies (Varshney et al., 2011; Singh et al., 2012) support the presence of gene families related to these functions, but they do not provide systematically curated inventories with traceable database accessions for individual waterlogging-responsive members. All entries represent putative homologs without waterlogging-specific expression or functional data in pigeonpea (Category III). Ortholog evidence from model species (*Arabidopsis*, rice, soybean) is classified as Category IV.

### 5.4 Transcription factor regulation

Transcription factors (TFs) are central regulators of gene expression during waterlogging stress, coordinating metabolic, physiological, and developmental responses to hypoxia. Genome-wide analyses have characterized the MYB (Yang et al., 2021), NAC (Singh et al., 2021), and AP2/ERF (Agarwal et al., 2016) transcription factor families in pigeonpea (**Table 5**). These TFs are proposed to regulate hypoxia-responsive genes involved in aerenchyma formation, programmed cell death, and other adaptive processes (**Figure 4**; Panel 4), but this proposal remains hypothetical in the absence of functional data.

**Table 5:** Major transcription factor families implicated in waterlogging tolerance in the pigeonpea genome.

Transcription factors (TFs) family	Role	Evidence in pigeonpea/(references)	Ortholog evidence (model species)/(references)
MYB	Ethylene, carbohydrate, antioxidant pathway regulation	Genome-wide R2R3-MYB family characterised in pigeonpea (Yang et al., 2021); waterlogging-specific functional members unidentified. Draft genome context: Varshney et al., 2011; Singh et al., 2012.	Hypoxia and secondary metabolism in Arabidopsis; drought/flooding in soybean (Hoeren et al., 1998; Kranz et al., 1998)
NAC	Hypoxia-ROS-hormone; PCD, aerenchyma	Genome-wide NAC family characterised across SAT legumes including pigeonpea (Singh et al., 2021); waterlogging-specific functional members unidentified. Draft genome context: Varshney et al., 2011; Singh et al., 2012.	Hypoxia-, senescence-, and flooding-responsive in Arabidopsis, soybean, rice (Nuruzzaman et al., 2010)
AP2/ERF	Hypoxia-responsive gene activation	Genome-wide AP2/ERF family characterised in pigeonpea and other legumes (Agarwal et al., 2016); waterlogging-specific responsive members and Group VII membership unverified. Draft genome context: Varshney et al., 2011; Singh et al., 2012.	ERF-VII Group: core oxygen-sensing regulators in Arabidopsis, rice, soybean. Group VII membership unverified in pigeonpea (Gibbs et al., 2011; Licausi et al., 2011; Gasch et al., 2016)

Genome-wide analyses are available for MYB (Yang et al., 2021), NAC (Singh et al., 2021) and AP2/ERF (Agarwal et al., 2016) in pigeonpea, but waterlogging-specific functional validation and Group VII ERF membership verification remain unavailable (Category III). Ortholog evidence from model species (*Arabidopsis*, rice, soybean) is classified as Category IV.

Critically, the principal oxygen-sensing mechanism in plants involves Group VII ERF transcription factors (ERF-VIIs), which are stabilized under hypoxia and activate core hypoxia-responsive genes via an evolutionarily conserved cis-motif (Gibbs et al., 2011; Gasch et al., 2016; Zubrycka et al., 2023) (Category IV). In Arabidopsis, this pathway is regulated by plant cysteine oxidases (PCOs) and the Arg/N-end rule proteolytic pathway (Licausi et al., 2011; Weits et al., 2014). While the pigeonpea genome undoubtedly contains AP2/ERF homologs, whether any represent functional Group VII members, whether PCO and N-end rule components regulate their stability, and whether this pathway operates during waterlogging in pigeonpea are entirely unknown.

### 5.5 Current status of molecular evidence

The molecular basis of waterlogging tolerance in pigeonpea remains at an early stage of discovery. The pigeonpea genome has been sequenced independently by two groups: Varshney et al. (2011) assembled approximately 72.7% of the 833 Mb genome of cultivar Asha (ICPL 87119), predicting 48,680 protein-coding genes, while Singh et al. (2012) assembled 510.8 Mb spanning 47,004 genes, including 152 associated with abiotic stress. Despite these genomic resources, functional validation of candidate genes under waterlogging conditions remains virtually absent. Large-scale polymorphism resources have been developed, including over 28,000 SNPs (Dubey et al., 2011; Upadhyaya et al., 2013), but these remain largely unconnected to phenotypic variation for waterlogging tolerance. No transgenic or CRISPR-based validation and no protein-level characterization currently exist for any candidate gene in *Cajanus cajan* under hypoxic conditions, beyond the single transcriptomic study discussed below (Tyagi et al., 2023).

### 5.6 Transcriptomic studies under waterlogging

Tyagi et al. (2023) examined two contrasting genotypes JBP-110B (tolerant) and ICP 7035 (susceptible) at the vegetative stage. One-month-old plants were subjected to waterlogging for 7 days (water level maintained at 5 cm above the soil surface), and leaf tissue was sampled at day 4 from control and stressed plants with 3 replications (**Figure 4**).

The *de novo* assembly yielded 90,084 non-redundant unigenes (N50 = 2,229 bp) after CD-HIT clustering, with 66,106 (73.3%) unigenes obtaining annotation from NR, GO, and KEGG databases. BUSCO assessment indicated 94.9% complete eukaryotic orthologs. The KEGG pathway enrichment analysis of “all the DEGs” was carried out using Omics Box. However, no specific differentially expressed gene counts, fold-change thresholds, false-discovery rates, lists of up- or down-regulated genes, or pathway enrichment statistics are reported. the GO and KEGG annotation displays the full unigene set rather than statistically documented differential expression between genotypes or treatments. We therefore consider the candidate genes from Tyagi et al. (2023) as a prioritized list for future validation, not as confirmed tolerance determinants.

Because the study does not report which specific genes or gene families (including ADH, PDC, SOD, APX, NAC, or ERF members) were identified as differentially expressed, transcriptome-derived candidate genes from this dataset cannot be classified as Category II evidence. The dataset remains a valuable hypothesis-generating resource Category II for the existence of a waterlogging transcriptome, Category III for specific gene claims but candidate genes require independent differential-expression analysis, appropriate biological replication, and functional validation before translation into breeding tools.

Similar limitations have been highlighted in other legume transcriptomic studies, emphasizing the need for functional validation before candidate genes can be translated into breeding targets. Transcriptome analysis between *Cajanus cajan* and its wild relative *Cajanus platycarpus* provided broader gene family inventories but did not specifically address waterlogging stress (Rathinam et al., 2019). The assembly yielded approximately 64,000 transcripts per species with 82% functional annotation, primarily matching *Glycine max*. While this resource expanded the genomic toolkit for pigeonpea, its relevance to waterlogging tolerance remains indirect.

Comparative studies in soybean provide independent support for several pathways identified in pigeonpea. Genome-wide prioritization of flooding-responsive genes identified conserved regulators associated with anaerobic metabolism, hormone signalling, ROS detoxification, and transcriptional regulation (Lai et al., 2021). Subsequent GWAS identified stable genomic regions associated with seed flooding tolerance (Sharmin et al., 2021), while QTL mapping further demonstrated that flooding tolerance is controlled by multiple loci with predominantly small phenotypic effects (Zhang et al., 2022). These studies suggest that many of the transcriptional responses observed in pigeonpea are biologically plausible; however, comparable genetic validation through GWAS, QTL mapping, or functional genomics is still lacking in pigeonpea.

### 5.7 Candidate genes identified through transcriptomics

Based on the available transcriptomic dataset (Tyagi et al., 2023) together with biochemical enzyme assays (Sairam et al., 2009), several putative candidate genes have been identified (**Table 6**). However, these genes should currently be regarded as hypothesis-generating candidates rather than confirmed determinants of waterlogging tolerance because none has been validated through independent transcriptomic studies, linkage mapping, genome-wide association studies, expression quantitative trait locus (eQTL) analysis, or genome editing in pigeonpea. We therefore consider the candidate genes from Tyagi et al. (2023) as a prioritized list for future validation, not as confirmed tolerance determinants.

**Table 6:** Candidate genes and pathways identified from transcriptome and biochemical studies in pigeonpea under waterlogging stress.

Gene/gene family	Putative function	Evidence type	Evidence category	Genotype	Tissue	Stage	Stress duration	Replicates	Supporting observation	Validation status
ADH	Ethanol fermentation, NAD^+^ regeneration	Biochemical enzyme assay	I	ICPL 84023, ICPL 20241, ICP 7035	Whole seedling/leaf	Vegetative	8 days	Not reported	Increased total ADH enzyme activity in tolerant genotypes (Kumutha et al., 2008; Bansal and Srivastava, 2015)	NV
ADH	Ethanol fermentation, NAD^+^ regeneration	Genome annotation	III	—	—	—	—	Not applicable	Genome-predicted only; no functional data under waterlogging in pigeonpea (Varshney et al., 2011; Singh et al., 2012)	NV
PDC	Pyruvate decarboxylation to acetaldehyde	Genome annotation	III	—	—	—	—	Not applicable	Genome-predicted only; no functional data under waterlogging in pigeonpea (Varshney et al., 2011; Singh et al., 2012)	NV
SOD	Superoxide dismutation	Biochemical enzyme assay	I	ICPL 84023 vs. susceptible	Whole seedling	Seedling	8 days	Not reported	Higher total SOD enzyme activity in tolerant genotype ICPL 84023 (Sairam et al., 2009)	NV
SOD	Superoxide dismutation	Genome annotation	III	—	—	—	—	Not applicable	Genome-predicted only; responsible isoforms unidentified (Varshney et al., 2011; Singh et al., 2012)	NV
CAT	H_2_O_2_ decomposition	Biochemical enzyme assay	I	ICPL 84023 vs. susceptible	Whole seedling	Seedling	8 days	Not reported	Higher total CAT enzyme activity in tolerant genotype (Sairam et al., 2009)	NV
APX	Ascorbate-mediated H_2_O_2_ reduction	Biochemical enzyme assay	I	ICPL 84023 vs. susceptible	Whole seedling	Seedling	8 days	Not reported	Higher total APX enzyme activity in tolerant genotype (Sairam et al., 2009)	NV

Tyagi et al. (2023) generated a *de novo* transcriptome from waterlogged leaf tissue of genotypes JBP-110B (tolerant) and ICP 7035 (susceptible), but the published Data Report does not identify specific differentially expressed genes, statistical thresholds, or enriched pathways. Consequently, ADH, NAC, ERF, and other gene families cannot be classified as Category II from this source. They remain Category III (genome-predicted only) unless supported by independent biochemical evidence (Category I for ADH, SOD, CAT, APX from Sairam et al., 2009; Kumutha et al., 2008; Bansal and Srivastava, 2015).

### 5.8 QTL and mapping studies

Genetic mapping for waterlogging tolerance in pigeonpea is extremely limited. Basavaraj et al. (2023) conducted genetic analysis of transient waterlogging tolerance in diverse pigeonpea genotypes, reporting morphological and biochemical trait associations. However, to date, no waterlogging-specific linkage or association mapping study in pigeonpea has produced validated QTL intervals, diagnostic markers, or estimates of phenotypic variance explained at the resolution required for marker-assisted breeding. Without high-resolution QTL mapping and its association with transcriptomic data, the bridge between phenotype and genotype remains incomplete.

### 5.9 Critical gaps in molecular evidence

Linked with soybean, where transcriptomics, GWAS, QTL mapping, and physiological analyses have together established a more comprehensive understanding of flooding tolerance (Sharmin et al., 2021; Zhang et al., 2022; Posso et al., 2023; Borella et al., 2014), molecular evidence in pigeonpea remains fragmentary. The current understanding relies primarily on a single RNA-seq dataset and a limited number of physiological studies. Consequently, transcriptome-derived candidate genes should be interpreted cautiously until supported by independent functional evidence. Second, expression quantitative trait locus (eQTL) studies have not been conducted, preventing the identification of regulatory variants underlying stress adaptation. Third, available enzyme assays measure total antioxidant activity without distinguishing isoform-specific functions or post-translational regulation (Sairam et al., 2009). Fourth, existing RNA-seq studies lack temporal resolution and isoform-specific analyses, limiting insights into dynamic transcriptional responses during waterlogging (Tyagi et al., 2023). Fifth, no validated QTL studies exist with sufficient marker density and phenotypic resolution to identify robust candidate genes or develop diagnostic markers. Finally, molecular evidence remains insufficient to capture the complex physiological and developmental responses to flooding.

### 5.10 A predictive integrative model for waterlogging tolerance in pigeonpea

Early sensing phase (**Figure 5A**): In model plants, restriction of gaseous diffusion due to root-zone hypoxia is thought to result in rapid depletion of oxygen levels. This is proposed to lead to influx of Ca^2+^ into the cytosol within hours and subsequent synthesis of ethylene, which may activate CDPK- and RBOH-dependent ROS bursts as secondary messengers (Category IV; Li et al., 2025; Yamauchi et al., 2017). At the same time, ERF-VII transcription factors are hypothesized to be stabilized by the N-end rule pathway, binding to hypoxia-responsive promoters (Gibbs et al., 2011; Giuntoli and Perata, 2017). In pigeonpea, these upstream sensors have not been tested and remain genome-predicted (Category III).

**Figure 5:**
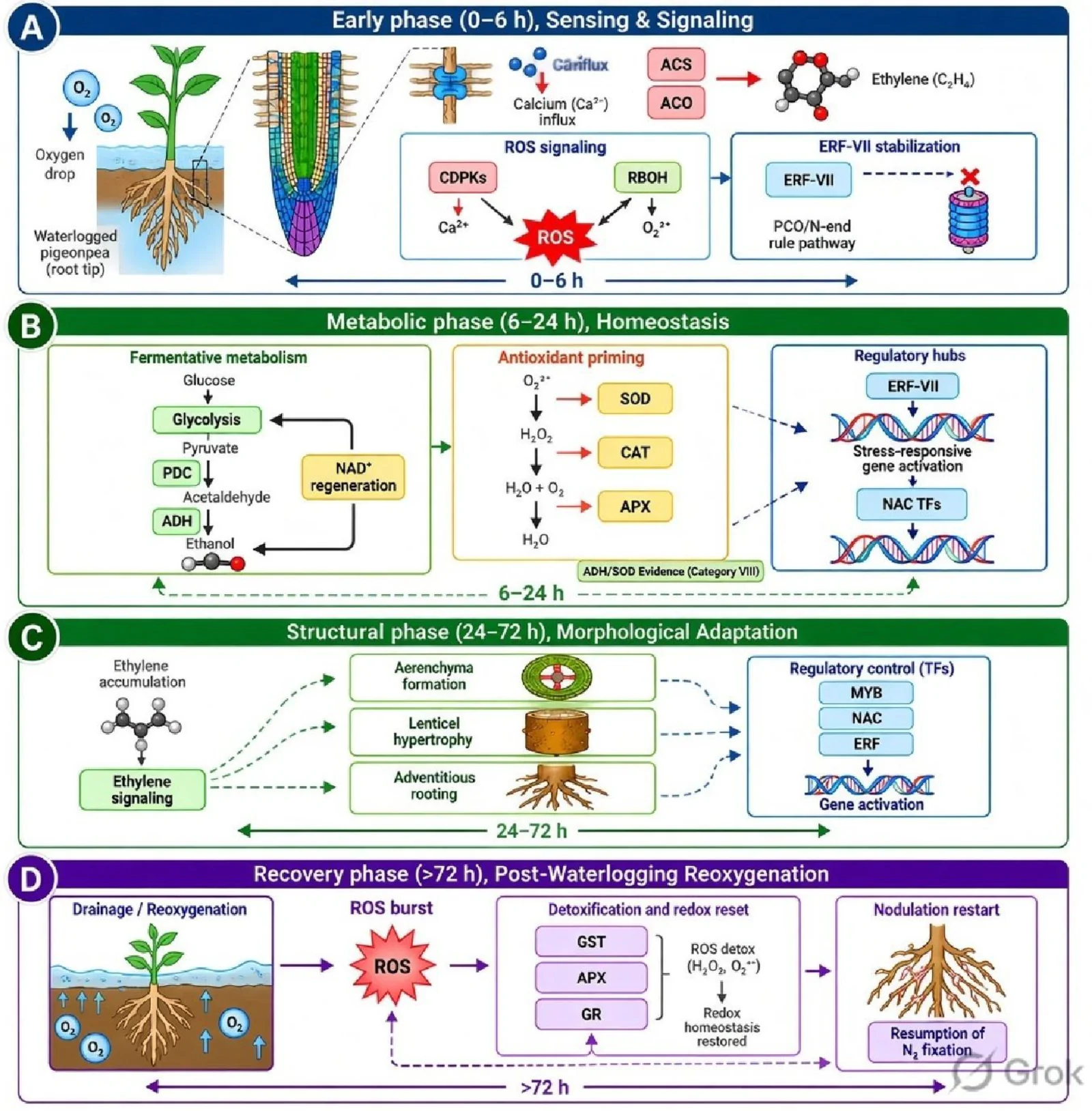
Temporal stress dynamics, physiological markers, metabolic pathways, and regulatory hubs: a provisional integrative model of waterlogging tolerance in pigeonpea derived from model species. The model is provisional and integrates validated pigeonpea-specific observations (Category I) with model-derived inference (Category IV). **(A)** Early sensing (0–6 h): in model plants, oxygen deprivation is thought to lead to influx of calcium, production of ethylene (ACS/ACO), and ROS signalling (CDPK/RBOH), resulting in stabilization of ERF-VII transcription factors through the PCO/N-end rule pathway. **(B)** Metabolic buffering (6–24 h): fermentative metabolism (PDC, ADH, LDH) and antioxidant priming (SOD, CAT, APX) maintain ATP and redox homeostasis. **(C)** Structural acclimation (24–72 h): internal O_2_ diffusion is restored by ethylene-induced aerenchyma formation, lenticel hypertrophy, and adventitious rooting. **(D)** Recovery (>72 h): post-drainage reoxygenation causes nodulation restart and ROS detoxification (GST, APX, GR). Hypothetical and model-inferred connections are shown by dashed arrows and are not validated in pigeonpea.

Metabolic buffering phase (**Figure 5B****):** When oxidative phosphorylation is compromised, cells switch to glycolysis and fermentation to continue generating ATP (Ashraf, 2012; Pan et al., 2021). ADH activity in tolerant pigeonpea genotypes is higher compared with susceptible genotypes (Bansal and Srivastava, 2015) (Category I). Antioxidant enzyme activities such as SOD, APX, and CAT were found to be higher in tolerant pigeonpea genotypes (Kumutha et al., 2009; Sairam et al., 2009) (Category I). The isoforms responsible and their transcriptional regulators have yet to be identified.

Structural acclimation phase (**Figure 5C**): Aerenchyma formation is hypothesized to involve ethylene-induced programmed cell death based on cross-species evidence (Category IV), though whether pigeonpea aerenchyma forms via lysigenous PCD, schizogenous separation, or both remains uncharacterized. Adventitious roots and hypertrophied lenticels develop and are associated with improved internal oxygen supply in tolerant genotypes (Category I) (Pan et al., 2021; Tiwari et al., 2024). For pigeonpea, the genetic control of these anatomical traits and their temporal coordination, which are reproducibly correlated with tolerance, are unknown (Belliappa et al., 2024; Hingane et al., 2015).

Recovery phase (**Figure 5D**): Upon drainage, reoxygenation triggers another ROS burst, and ROS scavenging systems are crucial for effective recovery (Kumutha et al., 2009; Yu et al., 2024). However, re-oxygenation causes secondary oxidative stress, which can be more damaging than hypoxia itself. Re-establishment of nodulation and nutrient uptake is also likely to play a significant role (Category V), which is yet to be systematically assessed in pigeonpea after drainage. We hypothesize that tolerant genotypes maintain higher rhizobial viability under hypoxia through maintaining oxygen transport to nodules, a prediction testable by nodule O_2_ imaging.

Predictive utility: The model makes testable predictions, including that early Ca^2+^/ethylene signatures should precede ADH/SOD upregulation in tolerant genotypes, that aerenchyma formation should correlate with ethylene-responsive NAC/ERF expression (Burke et al., 2020; Safavi-Rizi et al., 2020), and that genotypes lacking anatomical adaptations may compensate through constitutively higher fermentation or antioxidant capacity, as reported across waterlogging-tolerant crops (Ashraf, 2012; Sharma et al., 2021). These predictions need to be tested with time-course transcriptomics and high-resolution phenotyping.

### 5.11 Emerging dimensions: microbiome, metabolomics, and epigenetic regulation

While this review focuses on plant-intrinsic mechanisms, waterlogging tolerance is increasingly recognized as a multi-organismal and multi-omics trait.

Rhizosphere microbiome. Waterlogging shifts microbial communities from aerobic to facultative and anaerobic metabolisms, altering available nitrogen forms, ethylene precursors, and pathogen pressure. In soybean, specific *Bacillus* and *Pseudomonas* strains mitigate waterlogging damage by producing ACC deaminase, which cleaves the ethylene precursor ACC and reduces stress signal ling (Grichko and Glick, 2001). In pigeonpea, rhizobia-dependent symbiotic nitrogen fixation is waterlogging-susceptible (Pucciariello et al., 2019), yet no study has characterized whether tolerant genotypes recruit distinct microbial consortia or maintain rhizobial viability under hypoxia. This represents a major knowledge gap (Category V).

Metabolomics. Hypoxia triggers extensive reprogramming beyond ethanol and lactate fermentation, including the γ-aminobutyric acid (GABA) shunt, alanine accumulation, and shifts in the NADH/NAD^+^ ratio. In *Arabidopsis* and rice, these pathways act as metabolic “safety valves” that regenerate NAD^+^ while avoiding toxic lactate accumulation (Rocha et al., 2010). No metabolomic study has profiled pigeonpea under waterlogging; therefore, the metabolites that distinguish tolerant from susceptible genotypes remain unknown. Integrating metabolomics with transcriptomics (Tyagi et al., 2023) would help validate whether differentially expressed genes translate into predicted metabolic fluxes.

Epigenetic regulation. Transient waterlogging in maize and *Arabidopsis* induces DNA methylation changes and histone modifications that can prime plants for subsequent stress events (hypoxia memory). Because pigeonpea experiences intermittent monsoon flooding, epigenetic priming could be agronomically relevant. However, no study has examined chromatin remodelling, DNA methylation, or small RNA responses to hypoxia in *Cajanus*. We propose that multi-omics studies incorporating epigenomic and metabolomic layers should follow the transcriptomic benchmarks established by Tyagi et al. (2023).

## 6 Screening methodologies and genetic resources

### 6.1 Initial research efforts

Waterlogging has long been recognized as a major constraint in pigeonpea production, particularly in the semi-arid tropics. While soil and crop management practices such as raised sloping beds, ridge sowing, and transplanting of seedlings can reduce waterlogging damage, they are economically unfeasible for resource-poor farmers. Thus, the use of tolerant genotypes represents the most practical and cost-effective solution.

Early investigations highlighted that seedling establishment is the most critical stage for pigeonpea in waterlogging-prone environments (Khare et al., 2002). Biochemical studies subsequently provided mechanistic insights into tolerance. For example, experiments comparing the tolerant genotype ICPL 84023 and the susceptible ICP 7035 revealed that tolerance is associated with the ability to upregulate reactive oxygen species (ROS)-scavenging enzymes such as SOD, CAT, POD, and APX under waterlogging stress (Sairam et al., 2009). This was one of the first reports to link antioxidant defense capacity with waterlogging tolerance, extending screening criteria beyond purely morphological traits.

Parallel efforts investigated the genetic control of tolerance. Sarode et al. (2007) evaluated six standard generations (P_1_, P_2_, F_1_, F_2_, B_1_, and B_2_) derived from crosses involving ICPL 84023 (tolerant) and two susceptible genotypes (MA 98 PTH 1 and DA 11). Using the pot technique, seedlings were exposed to 8 days of waterlogging stress, and survival was recorded. Results demonstrated consistent survival in the tolerant parent, severe mortality in susceptible parents, and intermediate performance in F_1_s. Segregation in F_2_ and backcrosses indicated that tolerance is a monogenic trait and govern by a single dominant gene. This pioneering study provided the theoretical foundation for subsequent physiological and molecular investigations and underscored the importance of targeting seedling survival and qualitative gene action in breeding programs.

### 6.2 Screening protocols and physiological markers

Multi-tier screening protocols seed-submergence assays (192 h; Khare et al., 2002; Sultana et al., 2013), seedling-stage pot waterlogging (8–11 d from 15–35 DAS), and field validation in deep Vertisols have been used to identify tolerant genotypes during germination and early establishment. **Supplementary Table S1** inventories 23 evaluated genotypes, detailing the stress protocols, survival criteria, and key morpho-physiological traits recorded for each entry.

Tolerance markers include upregulated ROS-scavenging enzymes (SOD, CAT, POD, APX) in genotypes such as ICPL 84023 (Sairam et al., 2009), and anatomical adaptations (aerenchyma, hypertrophied lenticels, adventitious roots) in ICP 5028, ICPL 20241, and ICPH 2740 (Hingane et al., 2015).

Nevertheless, **Supplementary Table S1** highlights critical constraints: Most reported screening studies have focused on germination, seedling, or early vegetative responses, with limited systematic validation of tolerance through reproductive development and grain yield. Protocol heterogeneity in stress duration, water depth, and environment limits cross-study comparison, and genotype × environment interactions remain unquantified. Seedling survival should therefore not be treated as a proxy for reproductive-stage yield stability.

### 6.3 Recommended standardization of waterlogging screenin

The substantial heterogeneity among published screening protocols limits direct comparison of tolerance estimates across pigeonpea studies. Differences in plant developmental stage, water depth, stress duration, soil characteristics, environmental conditions, and recovery assessment can substantially alter the effective severity of waterlogging. A standardized framework should therefore define the stress environment and phenotyping criteria sufficiently to permit comparison among genotypes and experiments. Based on the available evidence and established principles of waterlogging phenotyping, the following parameters are proposed as a starting framework rather than as universally validated pigeonpea thresholds (**Table 7**; **Supplementary Table S1**).

**Table 7:** Summary of key studies screening the primary gene pool of pigeonpea for waterlogging tolerance (Evidence: Category I).

Germplasm evaluated	Screening stage/method	Tolerant donors	Susceptible checks	Major contribution/evidence strength (E.S)	References
Crosses (ICPL 84023 × susceptible lines); P1, P2, F1, F2, B1, B2 generations; 40-day-old seedlings; Kharif 2003–04	Pot technique; 8-day waterlogging at 3–6 cm water depth; seedling stage; plant survival scored 2 days after drainage	ICPL 84023	MA 98 PTH 1, DA 11	Proposed single dominant inheritance of waterlogging tolerance based on segregation analysis; tolerance governed by a single dominant gene (designated WR); 3:1 (tolerant:susceptible) segregation in F2; 1:1 in BC2; gene can be easily transferred by backcrossing/**E.S: Moderate**	Sarode et al., 2007
160 accessions total: 146 mini-core germplasm accessions + 4 control entries + 10 previously tested genotypes; 5 trials at 40, 50 DAS (2008) and 40, 62, 76 DAS (2009)	Seedling stage; temporary waterlogging; 5 field trials at 40–76 DAS; survival + recovery scored per trial	24 highly tolerant accessions; 39 tolerant accessions (none of the previously tested or control entries appeared in the highly tolerant group)	37 susceptible accessions including ICP 7035, ICP 8338, ICP 13562; 18 moderately susceptible; 42 moderately tolerant	First systematic evaluation of the pigeonpea mini-core germplasm for waterlogging tolerance; heritability estimated (0.27–0.75 per trial; 0.40 overall); hierarchical cluster analysis yielded five distinct tolerance groups/**E.S: High**	Krishnamurthy et al., 2012
272 diverse pigeonpea accessions (germplasm, elite lines, hybrids, commercial varieties)	Seed submergence assay (0, 120, 144, 168, 192 h); seedling-stage pot screening (96 genotypes); field evaluation (49 genotypes); three sequential levels of testing	Genotypes consistently tolerant across all three test levels: ICP 5028, ICPH 2740, ICPH 2671, ICPL 20992, MAL 9, LRG 30, Maruti, ICPL 20128, ICPL 332, ICPL 20237, ICPL 20238, ICPL 87119 (Asha), MAL 15	ICP 7035, ICP 8863	Developed and validated a three-stage screening protocol (*in vitro* seed submergence + seedling pot + field); standardized methodology; identified a large set of donors consistent across environments; hybrids showed higher average survival than germplasm accessions/**E.S: High**	Sultana et al., 2013
Selected waterlogging-tolerant and susceptible pigeonpea lines; morphophysiological characterization under controlled waterlogging stress	Morphological + anatomical + physiological analyses; pot waterlogging; transverse sections for aerenchyma and lenticel characterization	All 7 tolerant genotypes: ICP 5028, ICPH 2431, ICPL 87119, ICPH 2740, ICPL 149, ICPL 20241, MAL 15 Trait distribution: Aerenchyma — all 7 genotypes; Lenticels — ICP 5028, ICPH 2431, ICPL 87119, ICPH 2740, MAL 15 (5 genotypes); Adventitious roots — ICPL 149, ICPL 20241 (2 genotypes); No traits present in susceptible genotypes	ICP 7035 (susceptible; none of the anatomical traits formed)	First study to characterize anatomical adaptation mechanisms in pigeonpea under waterlogging; demonstrated that aerenchyma formation is universal in tolerant genotypes; lenticels and adventitious roots are differential — different tolerant genotypes use different morphological strategies; all traits absent in susceptible genotypes/**E.S: Moderate**	Hingane et al., 2015
Contrasting waterlogging-tolerant and susceptible pigeonpea genotypes under seedling-stage waterlogging stress	Seedling vigor traits: plumule length (PL) and radicle length (RL) under waterlogging; pot-based screening	ICP 5028, ICPL 20241, ICPH 2740	ICP 7035, ICPL 87051	Established seedling plumule and radicle length as quantitative selection criteria for waterlogging tolerance; demonstrated that higher survival is related to seed reserve and ease of imbibition through seed layers; supported ICP 5028, ICPL 20241, ICPH 2740 as consistent donors/**E.S: Moderate**	Kumar et al., 2020

Bold values indicate the evidence-strength (E.S.) classification reported for the corresponding study.

For controlled screening, waterlogging should be imposed at clearly defined developmental stages, with at least one early-stage assay and one reproductive-stage assay. Water depth should be maintained at a defined level relative to the soil surface, for example approximately 2–3 cm for seedlings and 5 cm for more developed plants. Rather than relying on a single exposure period, a duration gradient such as 4, 8, and 12 days could be used to characterize genotype-specific response curves. These durations should be regarded as proposed screening levels requiring validation rather than as fixed critical thresholds. Soil type, bulk density, initial moisture status, ambient and water temperature, and water depth should be standardized and explicitly reported. Where feasible, soil redox potential or dissolved oxygen should also be monitored because water depth alone does not fully capture the intensity of root-zone hypoxia.

Phenotyping should extend beyond survival during stress. Survival should be recorded both at the end of waterlogging and after a defined recovery period, preferably one week after drainage, and again after two weeks, to distinguish stress survival from post-stress recovery. Recovery should be based on predefined criteria such as continued survival, new leaf emergence, shoot regrowth, and restoration of plant vigor. For breeding applications, physiological measurements should ultimately be linked to reproductive performance, including flower and pod retention, seed production, seed weight, and grain yield. At least three independent biological replicates should be used where feasible, with randomized experimental layouts and appropriate estimates of experimental variance. Promising genotypes identified under controlled conditions should subsequently be evaluated across environments and seasons to quantify genotype × environment interactions.

## 7 Genetic resources for improving waterlogging tolerance in pigeonpea

The genetic resources available for improving waterlogging tolerance in pigeonpea range from cultivated germplasm to distantly related wild species (tertiary gene pool). These resources differ substantially in the extent of available genetic variation, crossability with cultivated pigeonpea, and ease of utilization in breeding programs. While cultivated germplasm offers immediate breeding utility, secondary and tertiary gene pool species provide novel adaptive alleles but are constrained by varying degrees of reproductive incompatibility. Therefore, the breeding value of each gene pool should be considered in terms of both genetic potential and practical feasibility.

### 7.1 Primary gene pool: cultivated pigeonpea

Screening of cultivated pigeonpea (2n =22) germplasm at seed and seedling stages has revealed substantial genetic variation in waterlogging tolerance. Early studies consistently classified genotypes into tolerant, moderately tolerant, and susceptible groups based on survival and senescence scores. Genotypes such as ICPL 20241, ICPH 2740, ICP 5028, MAL 15, and ICPL 87119 were repeatedly identified as tolerant donors, whereas ICP 7035 and ICP 8863 were consistently susceptible (Sultana et al., 2013; Hingane et al., 2015).

A major advance was achieved by Krishnamurthy et al. (2012), who evaluated the 146-accession pigeonpea mini-core collection representing global diversity. Significant variation in temporary waterlogging tolerance was observed, although only a small proportion (n=24≈ 16.4%) of accessions exhibited consistently high survival and recovery, indicating that favorable tolerance alleles are relatively rare within cultivated germplasm. Subsequently, Kumar et al. (2020) demonstrated that seedling vigor traits, particularly plumule length and root length, were positively associated with tolerance. Vigorous genotypes, including ICP 5028, ICPL 20241, and ICPH 2740, exhibited superior survival under waterlogging compared with weak seedlings such as ICP 7035 and ICPL 87051, highlighting seedling vigor as an additional selection criterion alongside anatomical adaptation (**Table 7**).

Although the primary gene pool provides the most readily exploitable source of waterlogging tolerance because identified tolerant accessions can be directly incorporated into conventional breeding programs without major reproductive barriers, the relatively low frequency of tolerant materials indicates that additional genetic variation may be required to achieve substantial genetic gains. These accessions are therefore potentially useful as donor parents for backcross-based breeding, but their effectiveness as donors should be validated across multiple elite genetic backgrounds and environments before wider breeding deployment.

### 7.2 Secondary gene pool: close wild relatives

Wild relatives in the secondary gene pool represent the most immediately accessible reservoir of adaptive alleles for pigeonpea improvement. All *Cajanus* species share the same diploid chromosome complement (2n = 22), which simplifies chromosome pairing in interspecific hybrids and underpins the feasibility of conventional introgression (Ohri and Singh, 2002; Varshney et al., 2011). Table 98summarizes the crossability, breeding barriers, and pre-breeding material status for each species.

Among secondary gene pool species, *C. acutifolius* is the most advanced in terms of pre-breeding deployment. Crosses with cultivated pigeonpea are moderately to highly successful, although partial sterility and reciprocal cross failure (when the wild parent is used as female) necessitate careful crossing design (Mallikarjuna and Saxena, 2002). Importantly, the hybridization barrier is surmountable without embryo rescue: F_1_ hybrids, BC_1_–BC_2_ families, and stable introgression lines (ILs) have been recovered through conventional backcrossing. Pre-breeding programs have already generated 149 BC_2_F_3_ introgression lines from the cross ICPL 87119 × ICPW 12, of which 12 agronomically superior ILs were selected for further evaluation (Pazhamala et al., 2015). Physiologically, these derivatives exhibit enhanced waterlogging/hypoxia tolerance associated with aerenchyma development, providing direct evidence that adaptive traits from *C. acutifolius* can be introgressed and functionally expressed in a cultivated background (Hingane et al., 2015). Furthermore, *C. acutifolius* is the source of the A_6_ cytoplasmic male sterility (CMS) system, which has been exploited to develop hybrid pigeonpea cultivars (Saxena and Kumar, 2003).

*C. sericeus* and *C. lineatus* are also classified within the secondary gene pool and have been reported as potential sources of waterlogging tolerance (Khoury et al., 2015; Pazhamala et al., 2015). Hybridization with *C. cajan* is moderately successful for both species, with F_1_ hybrids and derived lines recovered through standard crossing procedures (Saxena et al., 1996). The primary barriers are linkage drag in *C. sericeus* and reduced fertility in early backcross generations for *C. lineatus*. Neither species has undergone specific waterlogging evaluation in controlled experiments, so their tolerance potential remains putative. Nevertheless, pre-breeding materials are available.

### 7.3 Tertiary gene pool: distant wild relatives

The tertiary gene pool contains species with superior stress tolerance but substantially greater crossing barriers. *Cajanus platycarpus* and *C. crassus* have both been reported to survive prolonged flooding better than cultivated pigeonpea, exhibiting adaptive responses such as improved adventitious rooting and tolerance to prolonged hypoxia (Kassa et al., 2012; Rao et al., 2003; Belliappa et al., 2024). However, their practical utilization diverges sharply based on hybridization success (**Table 8**).

*C. platycarpus* is cross-compatible with cultivated pigeonpea only at very low frequency. Pre- and post-zygotic incompatibility cause embryo abortion within 6 days after pollination (DAP), and recovered F_1_ hybrids exhibit near-complete pollen sterility (Mallikarjuna and Moss, 1995). Consequently, embryo rescue is mandatory for hybrid recovery, and colchicine-induced chromosome doubling is required to restore fertility in the F_1_. Mallikarjuna and Moss (1995) first produced F_1_ hybrids via embryo rescue, and subsequent work recovered BC_4_F_1_ plants (Mallikarjuna, 2007). Colchicine-doubled tetraploid (4x) amphidiploids were also obtained, but these cannot be backcrossed directly to the diploid (2x) cultivated parent due to ploidy barriers, limiting their immediate utility (Mallikarjuna et al., 2011). Despite these constraints, *C. platycarpus* is the source of the A_7_ CMS cytoplasm, and its pre-breeding status is rated “established (with embryo rescue)” in **Table 8**. It’s very high waterlogging potential (surviving 21 days of pot waterlogging; Kassa et al., 2012) makes it a valuable long-term donor, provided that advanced tissue-culture and chromosome-manipulation protocols are applied.

**Table 8:** Wild relatives of pigeonpea (*Cajanus cajan*) with potential for waterlogging-tolerance introgression: gene pool, ploidy, cross-compatibility, breeding barriers, pre-breeding material status, and waterlogging potential.

Species	Gene pool	Ploidy (2n)	Crossability with C. cajan	Major breeding barriers	Hybrid/embryo rescue status	Available pre-breeding materials	Waterlogging potential/evidence strength	Evidence basis for rating
Cultivated donors (ICPL 20241, ICP 5028, ICPH 2740, etc.)	Primary	22	Excellent	None	Established (no intervention needed)	Extensive breeding lines, varieties, and CMS-based hybrids commercially deployed	High	Repeatedly validated across independent studies under laboratory, pot, and field conditions (Category I)
C. *acutifolius*	Secondary	22	High–Moderate	Partial sterility; reciprocal cross failure when wild parent is female	Established (F_1_ and backcross progeny recovered via conventional crossing)	F_1_ hybrids, BC_1_–BC_2_ families, and stable introgression lines (ILs) available; 149 BC_2_F_3_ lines developed from ICPL 87119 × ICPW 12, with 12 agronomically superior ILs selected; A_6_ CMS cytoplasm source	High	Interspecific derivatives showed enhanced tolerance with aerenchyma development (Hingane et al., 2015); pre-breeding populations validated (Pazhamala et al., 2015) (Category III)
C. *sericeus*	Secondary	22	Moderate–High	Linkage drag; low natural out-crossing (~2.3%)	Established (F_1_ hybrids and CMS lines recovered)	F_1_ hybrids produced; A_1_ CMS cytoplasm source; used in multi-parent crossing programs for pod borer resistance	Moderate	Reported as potential source of tolerance; no specific waterlogging evaluation published (Khoury et al., 2015; Saxena & Kumar, 2010) (Category I)
C. *lineatus*	Secondary	22	Moderate–High	Reduced fertility in early generations	Established (reciprocal crosses achieved; derived lines available)	F_1_ hybrids and derived lines (e.g., partial cleistogamous lines from C. cajan × C. lineatus); A_6_ CMS cytoplasm source	Moderate	Reported as potential source of tolerance; no specific waterlogging evaluation published (Pazhamala et al., 2015; Saxena et al., 1996) (Category I)
C. *platycarpus*	Tertiary	22	Very low	Embryo abortion within 6 DAP; post-zygotic incompatibility; F_1_ pollen sterility	Established (embryo rescue mandatory; colchicine doubling required)	F_1_ hybrids produced via embryo rescue (Mallikarjuna and Moss, 1995); colchicine-doubled tetraploid (4x) plants obtained but cannot be backcrossed to diploid (2x) cultivated parent; BC_4_F_1_ plants recovered (Mallikarjuna, 2007); A_7_ CMS cytoplasm source	Very high	Survived 21 days of pot waterlogging in greenhouse trials (Kassa et al., 2012); interspecific derivatives required embryo rescue (Mallikarjuna et al., 2011) (Category I)
C. *crassus*	Tertiary	22	Negligible	Severe pre- and post-zygotic incompatibility	None reported	No hybrids, embryo rescue protocols, or synthetic amphidiploids documented	Low	Reported to survive prolonged field flooding during monsoon in natural habitats (Rao et al., 2003); no successful hybridization with C. cajan documented (Category I)

All *Cajanus* species listed are diploid (2n = 22).

In contrast, *C. crassus* remains genetically inaccessible. Although it survives prolonged field flooding during the monsoon in its natural habitat (Rao et al., 2003) and possesses the same diploid chromosome number (2n = 22), no successful hybridization with *C. cajan* has been documented in the peer-reviewed literature. No embryo rescue protocol, F_1_ hybrid, backcross derivative, or synthetic amphidiploid has been reported.

The contrast between primary, secondary and tertiary gene pool species highlights a critical strategic decision for waterlogging-tolerance breeding (**Table 8**). These materials can enter variety development pipelines immediately. Tertiary gene pool species such as *C. platycarpus* require embryo rescue, chromosome doubling, and extensive backcrossing, meaning that only a limited number of introgression materials have been developed and linkage drag remains a major obstacle. *C. crassus* has not yet crossed the hybridization threshold at all. Therefore, for short- to medium-term variety development, breeders should prioritize cultivated donors and *C. acutifolius* derivatives, while treating *C. platycarpus* as a long-term pre-breeding resource and *C. crassus* as a target for future biotechnological intervention.

### 7.4 Key adaptive traits

Waterlogging-tolerant pigeonpea genotypes express several adaptive traits, including cortical aerenchyma, hypertrophied lenticels, adventitious roots, and high seedling vigor (Saxena et al., 2020; Hingane et al., 2015). Aerenchyma provides interconnected gas-filled spaces that facilitate longitudinal oxygen transport from shoots to submerged roots. Hypertrophied lenticels increase atmospheric oxygen entry at the root–shoot junction. Adventitious roots formed near the soil surface can replace oxygen-starved primary roots and may themselves contain aerenchyma, potentially sustaining water and nutrient uptake during prolonged flooding (Pan et al., 2021). These traits have been documented in tolerant genotypes including ICP 5028, ICPL 20241, and MAL 15 (Category I). We hypothesize that the carbon cost of aerenchyma formation may be offset by improved oxygen supply, but this trade-off has not been quantified in pigeonpea.

### 7.5 Role of seedling vigor and adaptive traits

Seedling vigor represents an important component of waterlogging tolerance. Traits such as plumule length and root length reflect the ability of seedlings to establish rapidly and withstand early flooding stress (Kumar et al., 2020). Vigorous genotypes, including ICP 5028, ICPL 20241, and ICPH 2740, consistently exhibited superior establishment and survival associated with hypertrophied lenticels and adventitious roots (Hingane et al., 2015), whereas susceptible genotypes such as ICP 7035 and ICPL 87051 showed poor seedling growth and reduced survival.

In contrast, some genotypes possess only a subset of adaptive traits. For example, MAL 15 develops hypertrophied lenticels despite relatively poor seedling vigor, suggesting greater reliance on post-germination morphological plasticity. Conversely, Asha and ICP 4924 exhibit tolerance through alternative mechanisms that remain to be fully characterized (Sultana et al., 2013). These observations indicate that different pigeonpea genotypes may utilize distinct combinations of adaptive traits, emphasizing the importance of multi-trait selection in breeding programs.

### 7.6 Reconciling phenotypic evidence across studies: stage dependence, recovery, and yield linkage

The evidence reviewed identifies recurrently reported seedling-stage tolerance-associated traits in pigeonpea; however, because much of this evidence derives from overlapping research groups, germplasm collections and experimental programmes, it does not yet constitute independent reproducibility across unrelated laboratories, environments and protocols. Systematic constraints therefore continue to limit translation into breeding tools. This subsection evaluates evidence strength, reconciles protocol discrepancies, distinguishes stress tolerance (survival during stress) from post-drainage recovery (ability to survive after stress removed), and assesses whether survival predicts yield.

Reproducibility and independence. Aerenchyma, hypertrophied lenticels, and adventitious roots have been reported in tolerant genotypes (ICP 5028, ICPL 20241, MAL 15) in more than one study (Hingane et al., 2015; Kumar et al., 2020). Because these reports originate partly from overlapping research groups and shared germplasm, they provide recurrent rather than fully independent confirmation, supporting moderate confidence that these anatomical traits are associated with seedling survival (Category I). Higher SOD, CAT, POD, and APX activities in ICPL 84023 relative to susceptible checks have also been reported (Sairam et al., 2009; Kumutha et al., 2009), although these studies likewise share germplasm and experimental lineage and so do not represent fully independent replication.

By contrast, transcriptome-derived candidates from Tyagi et al. (2023) rest on a single RNA-seq comparison of two genotypes at one vegetative-stage time point. Without isoform resolution, or functional validation, these genes remain Category II hypotheses. Prioritizing breeding targets requires distinguishing replicated Category I observations from unsupported Category II/III predictions.

Protocol heterogeneity and discrepancies across studies. Survival rankings vary because protocols differ in growth stage, water depth, duration, and recovery criteria. Seed-submergence assays (192 h at 23 °C; Sultana et al., 2013) test germination-stage hypoxia, whereas pot waterlogging (8–11 d; Hingane et al., 2015; Lal et al., 2017) imposes root-zone hypoxia on seedlings. Field validation relied on natural rainfall maintaining ~20 mm standing water from 7 DAS to more than month (Sultana et al., 2013), a condition impossible to replicate precisely across environments. These are not merely technical differences: a genotype surviving seed submergence may not tolerate seedling-stage soil waterlogging, and seedling survival does not imply reproductive-stage resilience.

Stress tolerance versus post-drainage recovery. Current screening focuses almost exclusively on survival during waterlogging (stress tolerance). Post-drainage recovery re-establishment of photosynthesis, root regeneration, nodulation restart, and detoxification of reoxygenation-induced ROS— has not been systematically evaluated. Reoxygenation triggers a secondary ROS burst that can exceed hypoxic injury (León et al., 2021). A genotype surviving hypoxia may fail to recover productivity if antioxidant reserves are depleted or nodule senescence is irreversible. Breeding programs should therefore integrate both stress-tolerance and recovery-capacity metrics, rather than assuming survival guarantees agronomic performance.

Survival and agronomic yield: an untested link. No study has rigorously quantified the correlation between seedling-stage survival and reproductive-stage grain yield under waterlogging in pigeonpea. Average annual yield losses of up to 30% have been reported across waterlogging stages in India (Sultana, 2010; Choudhary et al., 2011; Belliappa et al., 2024), but the contribution of specific tolerance traits to yield stability is unknown.

Complementary versus alternative strategies and carbon costs. Tolerant genotypes differ in trait combinations. ICP 5028, ICPH 2431, and MAL 15 develop aerenchyma with hypertrophied lenticels, whereas ICPL 149 and ICPL 20241 form aerenchyma and adventitious roots but lack lenticels (Hingane et al., 2015). Whether these represent complementary pathways or alternative strategies with different carbon costs is unresolved. Aerenchyma involves programmed cell death and cortical tissue loss; adventitious rooting diverts assimilates from shoot growth (Colmer and Voesenek, 2009). Whether structurally adapted genotypes pay yield penalties under non-stressed conditions has not been examined. Moreover, kiwifruit achieves tolerance through constitutively high fermentation and antioxidant capacity without aerenchyma (Gao et al., 2023), demonstrating that anatomical adaptations are not obligatory. Pigeonpea genotypes may therefore vary in their relative reliance on anatomical versus metabolic buffering. It must be emphasised, however, that no direct evidence currently quantifies the relative contributions of aerenchyma, lenticels, adventitious rooting, fermentation and antioxidant capacity, their associated carbon costs, or their consequences for yield. Whether these traits act as complementary or partially alternative strategies therefore remains a hypothesis that requires direct experimental testing under controlled, stage-specific conditions.

Absence of molecular–phenotypic bridges. Despite available genome assemblies, no validated QTLs, diagnostic markers, or functionally characterized candidate genes connect physiological traits to genomic loci in pigeonpea. The Group VII ERF oxygen-sensing pathway is established in Arabidopsis and rice (Category IV), but its operation in pigeonpea is speculative. Ethylene-mediated signalling, CDPK/RBOH-dependent ROS bursts, and NAC/ERF regulation are proposed from orthology (Category III/IV), yet none have been tested under hypoxia in pigeonpea through gene editing, transgenics, or expression QTL mapping. Short-read RNA-seq cannot resolve isoforms within multigene families (ADH, GST, NAC, AP2/ERF), leaving reported differentially expressed genes as putative candidates. This gap prevents deployment of marker-assisted selection or genomic prediction for waterlogging/hypoxia tolerance.

## 8 Translational breeding strategies and future priorities for waterlogging tolerance in pigeonpea

Conventional breeding remains the foundation of pigeonpea improvement, with tolerant donors such as ICP 5028, ICPL 20241, ICPH 2740, and MAL 15 providing valuable parental resources (Sultana et al., 2013; Hingane et al., 2015). Hypoxia tolerance is highly dependent on developmental stage, particularly early seedling establishment. Zaman et al. (2024) evaluated grass pea, field pea, lentil, and cowpea under waterlogging imposed at emergence and vegetative stages and found marked interspecific differences in tolerance. Grass pea exhibited consistent tolerance at both stages, whereas cowpea was susceptible during emergence but relatively tolerant during vegetative growth. These findings highlight the importance of incorporating stage-specific phenotyping into breeding programs and provide a useful comparative framework for developing waterlogging-tolerant pigeonpea genotypes.

The quantitative inheritance of waterlogging tolerance and strong genotype × environment interactions limit the efficiency of phenotypic selection alone. Future priorities should be addressed as a sequential pipeline rather than a list of technologies:

Priority 1 (1–3 Year): Standardized, stage-specific phenotyping. The immediate priority is development of standardized protocols that include germination, seedling, vegetative, and reproductive stages, with explicit assessment of recovery after drainage and yield under waterlogging. Future experiments should evaluate promising donors across multiple developmental stages, particularly flowering and pod-filling, using standardized and field-relevant waterlogging regimes. Measurements should extend beyond survival to include recovery, reproductive traits, grain yield, yield components, and harvest index. Multi-environment testing under contrasting soil-drainage and rainfall conditions, coupled with quantitative analysis of genotype × environment interactions, is required to identify donors with stable yield performance rather than merely high seedling survival. Such stage-specific, multi-environment validation would provide the essential phenotypic foundation for subsequent QTL mapping, genomic prediction, marker-assisted selection, and deployment of waterlogging-tolerant pigeonpea cultivars. Protocols should specify water depth, duration, soil type, temperature, and recovery period to enable cross-study comparison.

Priority 2 (Short-medium term, 3–5 Year): Multi-environment evaluation and heritability estimation. Replicated evaluation across multiple environments is needed to quantify genotype × environment interactions and estimate heritability for waterlogging tolerance traits. This is essential before deploying genomic selection or marker-assisted breeding.

Priority 3 (Short-medium term, 3–5 Year): Development of mapping populations. Biparental and multi-parent mapping populations should be developed from well-characterized tolerant and susceptible parents (e.g., ICPL 84023 × ICP 7035; ICP 5028 × ICPL 87051) and phenotyped under standardized waterlogging protocols. These parental combinations require independent validation of segregation and phenotyping rigor before they can be regarded as robust mapping resources (Category V).

Priority 4 (Medium term, 5–7 Year): Identification and independent validation of stable loci. High-density linkage mapping and genome-wide association studies, combined with the standardized phenotyping above, are needed to identify robust QTLs and associated markers. These must be validated in independent populations and environments before deployment.

Priority 5 (Medium-long term, 7–10 Year): Development of diagnostic markers. Once stable loci are validated, high-throughput SNP markers (e.g., KASP assays) can be developed for marker-assisted backcrossing (MABC) to introgress favorable alleles into elite cultivars while minimizing linkage drag (Collard and Mackill, 2008; Varshney et al., 2021).

Priority 6 (Medium-long term, 7–10 Year): Genomic selection and speed breeding. Genomic selection (GS) offers promise for improving this complex quantitative trait by associating genome-wide SNP markers with multi-environment phenotypic data to predict genomic breeding values without requiring prior identification of all causal loci (Crossa et al., 2017). Speed breeding with controlled-environment waterlogging screening could further shorten breeding cycles by enabling rapid generation advancement before field evaluation (Watson et al., 2018; Hickey et al., 2019).

Priority 7 (long term, > 10 Year): Functional validation through genome editing. Candidate genes identified through comparative genomics, including ADH, ERF-VII, CDPK, RBOH, NAC, ACS, ACO, and phytoglobins, represent promising targets for functional validation. However, CRISPR/Cas-mediated approaches should only be pursued once robust causal candidates have been identified, reliable phenotyping protocols are established, and efficient transformation systems are available for pigeonpea (Chen et al., 2019; Belliappa et al., 2024).

The proposed timelines should be interpreted as research horizons rather than fixed implementation deadlines or statements of current funding status. They reflect the logical dependency among activities: standardized phenotyping and reproductive-stage validation should precede genetic dissection; genetic associations should be followed by functional validation; and molecular deployment should be based on experimentally validated mechanisms. In particular, genome editing represents a longer-term strategy rather than an immediate breeding priority. Genome editing should be pursued only after robust phenotyping protocols and efficient transformation systems are established for pigeonpea and candidate genes have been validated using complementary genetic, physiological, molecular, or functional approaches.

Wild *Cajanus* species remain valuable reservoirs of novel adaptive alleles, but their utilization requires gene pool-specific breeding strategies. Secondary gene pool species are relatively amenable to hybridization, whereas tertiary species such as *C. platycarpus* and *C. crassus* require embryo rescue, *in vitro* embryo culture, and repeated backcrossing because of strong reproductive barriers (Mallikarjuna et al., 2006, 2011). Genomic-assisted introgression with marker-assisted background selection will facilitate the efficient utilization of these valuable wild resources while minimizing linkage drag (**Figure 6**).

**Figure 6:**
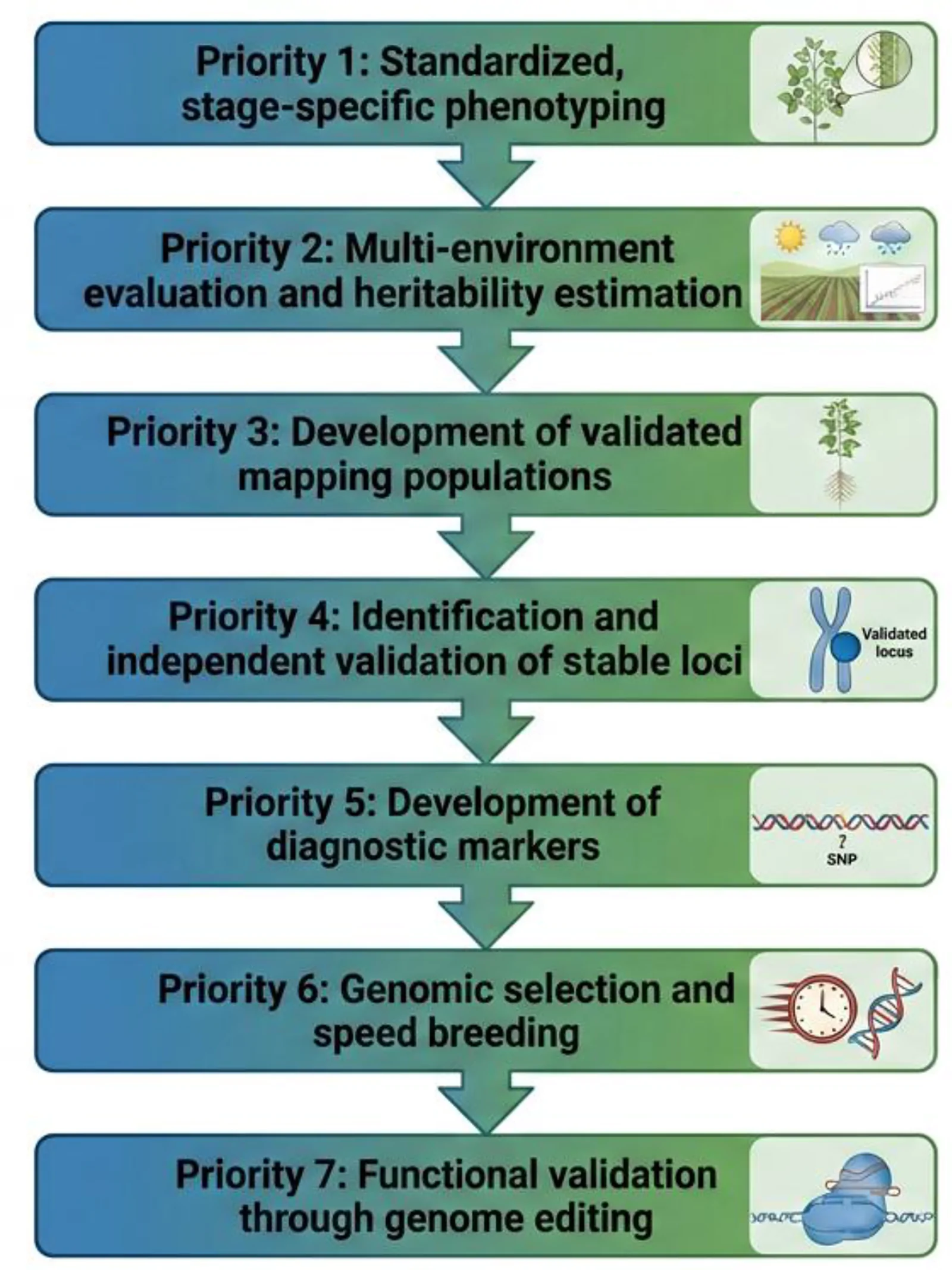
Sequential priority pipeline for translating waterlogging tolerance research into climate-resilient pigeonpea cultivars. The framework progresses from foundational phenotyping (Priority 1) through multi-environment validation and genetic mapping (Priorities 2–4), to molecular breeding tool development (Priority 5), genomic-assisted selection (Priority 6), and finally functional validation of causal candidates and genome editing should be pursued only after robust phenotyping protocols and efficient transformation systems are established for pigeonpea (Priority 7). This stepwise approach places standardized stage-specific phenotyping, recovery assessment, and independent locus validation as prerequisites for downstream genomic selection, marker deployment, and genome editing (Evidence: Category V).

Despite the availability of genome assemblies and SNP resources, molecular evidence for waterlogging tolerance in pigeonpea remains limited. To date, Tyagi et al. (2023) represents the only transcriptome-wide study investigating waterlogging-responsive gene expression, and none of the reported candidate genes has been validated through QTL mapping, analysis, transgenic approaches, or genome editing. Short-read RNA sequencing may have limited capacity to distinguish closely related paralogues and transcript isoforms within multigene families such as ADH, GST, NAC, and AP2/ERF; consequently, the reported genes should be regarded as putative candidates rather than validated determinants of hypoxia tolerance.

Waterlogging tolerance studies in soybean demonstrate that integrating RNA-seq, GWAS, QTL mapping, and physiological analyses provides substantially stronger evidence for flooding-tolerance pathways, including anaerobic fermentation, ROS detoxification, oxygen sensing, and post-flood recovery (Borella et al., 2014; Lai et al., 2021; Sharmin et al., 2021; Zhang et al., 2022; Posso et al., 2023). Future research in pigeonpea should therefore prioritize standardized multi-environment phenotyping, time-course transcriptomics, long-read sequencing, the integration of GWAS with transcriptomic data to prioritise candidate genes (noting that eQTL mapping is a distinct approach, requiring transcript-abundance phenotypes measured across an appropriately genotyped population and statistical mapping of the loci that control transcript abundance), and CRISPR/Cas-mediated functional validation to establish causal gene–trait relationships and accelerate genomic-assisted breeding.

## 9 Conclusions

Waterlogging is an increasingly important constraint to pigeonpea production under erratic rainfall and inadequate soil drainage. The evidence reviewed indicates that waterlogging tolerance is a complex, quantitative, and strongly stage-dependent trait involving coordinated anatomical, physiological, biochemical, and molecular responses rather than a single tolerance mechanism. A central conclusion of this review is the need to distinguish experimentally demonstrated responses in pigeonpea from mechanisms inferred from other species. In pigeonpea, aerenchyma formation, enhanced antioxidant activity, and increased fermentative responses have been documented in particular genotypes under defined experimental conditions and are associated with improved survival (Categories I–II). In contrast, regulatory mechanisms such as ethylene-mediated control of aerenchyma formation remain largely inferred from model and crop species including Arabidopsis and rice (Category IV). Thus, the current evidence supports associations between individual traits and waterlogging tolerance under specific conditions, but does not yet establish an integrated, experimentally validated causal model of tolerance in pigeonpea.

Phenotypic screening has identified promising donor resources, including ICPL 20241, ICP 5028, ICPH 2740, and MAL 15. However, their practical value for breeding should be interpreted in relation to the developmental stage and environment in which tolerance was demonstrated. The major unresolved issue is the limited validation of tolerance through reproductive development and its relationship with grain yield. Seedling survival should therefore not be considered a surrogate for reproductive-stage tolerance or yield stability. Future screening should evaluate promising donors and breeding lines at both early and reproductive stages under standardized, field-relevant waterlogging regimes, with post-drainage recovery, yield components, and grain yield assessed as distinct phenotypes.

The principal limitations of the current evidence base include heterogeneous waterlogging protocols, limited genotype diversity in molecular studies, inadequate reproductive-stage and yield validation, incomplete understanding of post-stress recovery, limited quantification of genotype × environment interactions, and the absence of functional validation for most candidate genes. These limitations constrain direct comparison among studies and currently limit the translation of physiological and transcriptomic observations into robust molecular breeding tools.

Accordingly, the immediate priority is not the premature deployment of molecular breeding technologies, but the establishment of a standardized and agronomically relevant phenotyping framework. Such a framework should specify water depth, stress duration, developmental stage, soil and environmental conditions, and post-drainage recovery, while incorporating reproductive performance and grain yield together with estimates of heritability and genotype × environment interaction across multiple environments. This should be followed by validation of promising donors through replicated crossing populations and stage-specific phenotyping to identify stable loci associated with agronomically meaningful tolerance.

Once stable phenotypes and reproducible loci have been established, QTL mapping, diagnostic marker development, genomic selection, speed breeding, and multi-omics approaches can be systematically integrated into breeding pipelines. Functional validation of candidate genes should precede their use as mechanistic targets for genome editing or other precision-breeding approaches. The strategic use of wild *Cajanus* relatives should likewise be guided by validated phenotypes and characterized loci. A phenotype-first, validation-driven pipeline—from standardized screening and reproductive-stage yield validation to genetic dissection, functional validation, and molecular deployment—offers the most defensible route toward climate-resilient pigeonpea cultivars. This framework may also provide a broader strategy for improving hypoxia and waterlogging tolerance in other grain legumes exposed to increasingly erratic rainfall and flooding.

## References

[B1] Agarwal G. Garg V. Kudapa H. Doddamani D. Pazhamala L. T. Khan A. W. . (2016). Genome-wide dissection of AP2/ERF and HSP90 gene families in five legumes and expression profiles in chickpea and pigeonpea. Plant Biotechnol. J. 14, 1563–1577. doi: 10.1111/pbi.12520 26800652 PMC5066796

[B2] Ahmed F. Rafii M. Y. Ismail M. R. Juraimi A. S. Rahim H. A. Asfaliza R. . (2013). Waterlogging tolerance of crops: Breeding, mechanism of tolerance, molecular approaches, and future prospects. BioMed. Res. Int. 2013, 963525. doi: 10.1155/2013/963525 23484164 PMC3591200

[B3] Ashraf M. A. (2012). Waterlogging stress in plants: A review. Afr. J. Agric. Res. 7, 1976–1981. doi: 10.5897/AJARX11.084

[B4] Bailey-Serres J. Fukao T. Gibbs D. J. Holdsworth M. J. Lee S. C. Licausi F. . (2012). Making sense of low oxygen sensing. Trends Plant Sci. 17, 129–138. doi: 10.1016/j.tplants.2011.12.004 22280796

[B5] Bailey-Serres J. Voesenek L. A. C. J. (2008). Flooding stress: Acclimations and genetic diversity. Annu. Rev. Plant Biol. 59, 313–339. doi: 10.1146/annurev.arplant.59.032607.092752 18444902

[B6] Bansal R. Srivastava J. P. (2015). Effect of waterlogging on photosynthetic and biochemical parameters in pigeonpea. Russ. J. Plant Physiol. 62, 322–327. doi: 10.1134/s1021443715030036

[B7] Basavaraj P. S. Rane J. Boraiah K. M. Gangashetty P. Harisha C. B. (2023). Genetic analysis of tolerance to transient waterlogging stress in pigeonpea (*Cajanus cajan* L. Millspaugh). Indian J. Genet. Plant Breed. 83, 316–325. doi: 10.31742/isgpb.83.3.3

[B8] Belliappa S. H. Bomma N. Pranati J. Soregaon C. D. Hingane A. Basavaraj P. S. . (2024). Breeding for waterlogging tolerance in pigeonpea: current status and future prospects. CABI Agric. Biosci. 5, 299. doi: 10.1186/s43170-024-00299-y38164791

[B9] Borella J. Amarante L. Oliveira D. S. C. Oliveira A. C. B. Braga E. J. B. (2014). Waterlogging-induced changes in fermentative metabolism in roots and nodules of soybean genotypes. Sci. Agric. 71, 499–508. doi: 10.1590/0103-9016-2014-004441099703

[B10] Burke R. Schwarze J. Sherwood O. L. Jnaid Y. McCabe P. F. Kacprzyk J. (2020). Stressed to death: The role of transcription factors in plant programmed cell death induced by abiotic and biotic stimuli. Front. Plant Sci. 11, 1235. doi: 10.3389/fpls.2020.01235 32903426 PMC7434935

[B11] Chauhan Y. S. Silim S. N. Kumar Rao J. V. D. K. Johansen C. (1997). A pot technique to screen pigeonpea cultivars for resistance to waterlogging. J. Agron. Crop Sci. 178, 179–183. doi: 10.1111/j.1439-037x.1997.tb00487.x40046247

[B12] Chen K. Wang Y. Zhang R. Zhang H. Gao C. (2019). CRISPR/Cas genome editing and precision plant breeding in agriculture. Annu. Rev. Plant Biol. 70, 667–697. doi: 10.1146/annurev-arplant-050718-100049 30835493

[B14] Choudhary A. K. Sultana R. Pratap A. Nadarajan N. Jha U. C. (2011). Breeding for abiotic stresses in pigeonpea. J. Food. Legumes 24, 165–174.

[B15] Cojean A. N. Y. Lehmann M. F. Robertson E. K. Thamdrup B. Zopfi J. (2020). Controls of H_2_S, Fe^2+^, and Mn^2+^ on microbial NO_3_^-^-reducing processes in sediments of an eutrophic lake. Front. Microbiol. 11, 1158. doi: 10.3389/fmicb.2020.01158 32612583 PMC7308436

[B16] Collard B. C. Y. Mackill D. J. (2008). Marker-assisted selection: An approach for precision plant breeding in the twenty-first century. Philos. Trans. R. Soc. B. Biol. Sci. 363, 557–572. doi: 10.1098/rstb.2007.2170 17715053 PMC2610170

[B17] Colmer T. D. Voesenek L. A. C. J. (2009). Flooding tolerance: suites of plant traits in variable environments. Funct. Plant Biol. 36, 665–681. doi: 10.1071/FP09144 32688679

[B18] Crossa J. Pérez-Rodríguez P. Cuevas J. Montesinos-López O. Jarquín D. de los Campos G. . (2017). Genomic selection in plant breeding: Methods, models, and perspectives. Trends Plant Sci. 22, 961–975. doi: 10.1016/j.tplants.2017.08.011 28965742

[B19] Dixon D. P. Skipsey M. Edwards R. (2010). Roles for glutathione transferases in plant secondary metabolism. Phytochemistry 71, 338–350. doi: 10.1016/j.phytochem.2009.12.012 20079507

[B20] Dubey A. Farmer A. Schlueter J. Cannon S. B. Abernathy B. Tuteja R. . (2011). Defining the transcriptome assembly and its use for genome dynamics and transcriptome profiling studies in pigeonpea [Cajanus cajan (L.) Millsp. DNA Res. 18, 153–164. doi: 10.1093/dnares/dsr007 21565938 PMC3111231

[B21] Ejiri M. Fukao T. Miyashita T. Shiono K. (2021). A barrier to radial oxygen loss helps the root system cope with waterlogging-induced hypoxia. Breed. Sci. 71, 40–50. doi: 10.1270/jsbbs.20110 33762875 PMC7973497

[B22] FAOSTAT (2023). Fao Statistical Database (Rome: Food and Agriculture Organization of the United Nations).

[B23] Fukao T. Barrera-Figueroa B. E. Juntawong P. Peña-Castro J. M. (2019). Submergence and waterlogging stress in plants: A review highlighting research opportunities and understudied aspects. Front. Plant Sci. 10, 340. doi: 10.3389/fpls.2019.00340 30967888 PMC6439527

[B24] Gao M. Gai C. Li X. Feng X. Lai R. Song Y. . (2023). Waterlogging tolerance of Actinidia valvata Dunn is associated with high activities of pyruvate decarboxylase, alcohol dehydrogenase and antioxidant enzymes. Plants 12, 2872. doi: 10.3390/plants12152872 37571025 PMC10421509

[B25] Gasch P. Fundinger M. Müller J. T. Lee T. Bailey-Serres J. Mustroph A. (2016). Redundant ERF-VII transcription factors bind to an evolutionarily conserved cis-motif to regulate hypoxia-responsive gene expression in Arabidopsis. Plant Cell 28, 160–180. doi: 10.1105/tpc.15.00866 26668304 PMC4746684

[B26] Gibbs D. J. Lee S. C. Isa N. M. Gramuglia S. Fukao T. Bassel G. W. . (2011). Homeostatic response to hypoxia is regulated by the N-end rule pathway in plants. Nature 479, 415–418. doi: 10.1038/nature10534 22020279 PMC3223408

[B27] Gill S. S. Tuteja N. (2010). Reactive oxygen species and antioxidant machinery in abiotic stress tolerance in crop plants. Plant Physiol. Biochem. 48, 909–930. doi: 10.1016/j.plaphy.2010.08.016 20870416

[B28] Giuntoli B. Perata P. (2017). Group VII ethylene response factors in arabidopsis: regulation and physiological roles. Plant Physiol. 176, 1143–1155. doi: 10.1104/pp.17.01225 29269576 PMC5813551

[B29] Grichko V. P. Glick B. R. (2001). Amelioration of flooding stress by ACC deaminase-containing plant growth-promoting bacteria. Plant Physiol. Biochem. 39, 11–17. doi: 10.1016/S0981-9428(00)01212-2

[B30] Hasanuzzaman M. Bhuyan M. H. M. B. Zulfiqar F. Raza A. Mohsin S. M. Mahmud J. A. . (2020). Reactive oxygen species and antioxidant defense in plants under abiotic stress: revisiting the crucial role of a universal defense regulator. Antioxidants 9, 681. doi: 10.3390/antiox9080681 32751256 PMC7465626

[B31] Hickey L. T. Hafeez A. N. Robinson H. Jackson S. A. Leal-Bertioli S. C. M. Tester M. . (2019). Breeding crops to feed 10 billion. Nat. Biotechnol. 37, 744–754. doi: 10.1038/s41587-019-0152-9 31209375

[B32] Hingane A. Saxena K. B. Patil S. B. Sultana R. Srikanth S. Mallikarjuna N. . (2015). Mechanism of waterlogging tolerance in pigeonpea. Indian J. Genet. Plant Breed. 75, 208. doi: 10.5958/0975-6906.2015.00032.2

[B33] Hoeren F. U. Dolferus R. Wu Y. Peacock W. J. Dennis E. S. (1998). Evidence for a role for AtMYB2 in the induction of the Arabidopsis alcohol dehydrogenase gene (ADH1) by low oxygen. Genetics 149, 479–490. doi: 10.1093/genetics/149.2.479 9611167 PMC1460183

[B34] Ismond K. P. Dolferus R. De Pauw M. Dennis E. S. Good A. G. (2003). Enhanced low oxygen survival in Arabidopsis through increased metabolic flux in the fermentative pathway. Plant Physiol. 132, 1292–1302. doi: 10.1104/pp.103.022244 12857811 PMC167069

[B35] Jackson M. B. Armstrong W. (1999). Formation of aerenchyma and the processes of plant ventilation in relation to soil flooding and submergence. Plant Biol. 1, 274–287. doi: 10.1111/j.1438-8677.1999.tb00253.x40046247

[B36] Kassa M. T. Penmetsa R. V. Carrasquilla-Garcia N. Sarma B. K. Datta S. Upadhyaya H. D. . (2012). Genetic patterns of domestication in pigeonpea [Cajanus cajan (L.) Millsp.] and wild Cajanus relatives. PloS One 7, e39563. doi: 10.1371/journal.pone.0039563 22745789 PMC3382124

[B37] Khare D. Rao S. Lakhani J. P. Satpute R. G. (2002). Tolerance for flooding during germination in pigeonpea. Seed Res. 30, 82–87.

[B38] Khoury C. K. Castañeda-Álvarez N. P. Achicanoy H. A. Sosa C. C. Bernau V. Kassa M. T. . (2015). Crop wild relatives of pigeonpea [Cajanus cajan (L.) Millsp.]: Distributions, ex situ conservation status, and potential genetic resources for abiotic stress. Crop Sci. 55, 1–14. doi: 10.1016/j.biocon.2015.01.03242574925

[B39] Klok E. J. Wilson I. W. Wilson D. Chapman S. C. Ewing R. M. Somerville S. C. . (2002). Expression profile analysis of the low-oxygen response in Arabidopsis root cultures. Plant Cell 14, 2481–2494. doi: 10.1105/tpc.004747 12368499 PMC151230

[B40] Komatsu S. Deschamps T. Hiraga S. Kato M. Chiba M. Hashiguchi A. . (2011). Characterization of a novel flooding stress-responsive alcohol dehydrogenase expressed in soybean roots. Plant Mol. Biol. 77, 309–322. doi: 10.1007/s11103-011-9812-y 21811849

[B1212] Kranz H. D. Denekamp M. Greco R. Jin H. Leyva R. Meissner R. C. . (1998). Towards functional characterisation of the members of the R2R3-MYB gene family from *Arabidopsis thaliana*. The Plant Journal 16 (2), 263–276. doi: 10.1046/j.1365-313x.1998.00278.x 9839469

[B41] Krishnamurthy L. Upadhyaya H. D. Saxena K. B. Vadez V. (2012). Variation for temporary waterlogging response within the mini core pigeonpea germplasm. J. Agric. Sci. 150, 357–364. doi: 10.1017/S002185961100068241292463

[B42] Kumar C. V. S. Naik S. J. S. Mohan N. Saxena R. K. Varshney R. K. (2017). “ Botanical description of pigeonpea [Cajanus cajan (L.) millsp,” in Compendium of Plant Genomes. Ed. Springer ( Springer, Cham), 17–29. doi: 10.1007/978-3-319-63797-6_3

[B43] Kumar D. Sultana R. Kumar R. R. Kirti M. (2020). Characterization of pigeonpea genotypes for waterlogging tolerance based on morpho-physiological and molecular traits. Curr. J. Appl. Sci. Technol. 39, 21–33. doi: 10.9734/cjast/2020/v39i123066042706982

[B44] Kumutha D. Ezhilmathi K. Sairam R. K. Srivastava G. C. Deshmukh P. S. Meena R. C. (2009). Waterlogging induced oxidative stress and antioxidant activity in pigeonpea genotypes. Biol. Plant 53, 75–84. doi: 10.1007/s10535-009-0011-530311153

[B45] Kumutha D. Sairam R. K. Ezhilmathi K. Chinnusamy V. Meena R. C. (2008). Effect of waterlogging on carbohydrate metabolism in pigeonpea [Cajanus cajan (L.) Millsp.]: Upregulation of sucrose synthase and alcohol dehydrogenase. Plant Sci. 175, 706–716. doi: 10.1016/j.plantsci.2008.07.01342574925

[B46] Lai M. C. Lai Z. Y. Jhan L. H. Lai Y. S. Kao C. F. (2021). Prioritization and evaluation of flooding tolerance genes in soybean (Glycine max L. Merr.). Front. Genet. 11, 612131. doi: 10.3389/fgene.2020.612131 33584812 PMC7873447

[B47] Lal M. Kumar A. Jangra M. Kumar R. Sheokand S. (2018). Screening of pigeonpea [Cajanus cajan (L.) Millsp.] genotypes for waterlogging, salinity and combined waterlogging and salinity tolerance. Int. J. Pure Appl. Bioscience 6, 128–136. doi: 10.18782/2320-7051.6262

[B48] Lal M. Sangwan A. Kumar P. Sheokand S. (2017). Effects of waterlogging, salinity and their combination on seed germination and vigour index in pigeonpea. Int. J. Curr. Microbiol. Appl. Sci. 6, 945–951. doi: 10.20546/ijcmas.2017.612.104

[B49] Langan P. Bernád V. Walsh J. Henchy J. Khodaeiaminjan M. Mangina E. . (2022). Phenotyping for waterlogging tolerance in crops: Current trends and future prospects. J. Exp. Bot. 73, 5149–5169. doi: 10.1093/jxb/erac243 35642593 PMC9440438

[B50] León J. Castillo M. C. Gayubas B. (2021). The hypoxia-reoxygenation stress in plants. J. Exp. Bot. 72, 5841–5856. doi: 10.1093/jxb/eraa591 33367851 PMC8355755

[B51] Li J. Ishii T. Yoshioka M. Hino Y. Nomoto M. Tada Y. . (2025). CDPK5 and CDPK13 play key roles in acclimation to low oxygen through the control of RBOH-mediated ROS production in rice. Plant Physiol. 197, kiae293. doi: 10.1093/plphys/kiae293 38849987 PMC11663579

[B52] Licausi F. Kosmacz M. Weits D. A. Giuntoli B. Giorgi F. M. Voesenek L. A. C. J. . (2011). Oxygen sensing in plants is mediated by an N-end rule pathway for protein destabilization. Nature 479, 419–422. doi: 10.1038/nature10536 22020282

[B53] Loreti E. Valeri M. C. Novi G. Perata P. (2018). Gene regulation and survival under hypoxia requires starch availability and metabolism. Plant Physiol. 176, 1286–1298. doi: 10.1104/pp.17.01002 29084901 PMC5813553

[B54] Mallikarjuna N. (2007). Production of fertile progeny from interspecific incompatible cross Cajanus platycarpus × C. cajan. J. SAT Agric. Res. 3, 1–3.

[B55] Mallikarjuna N. Jadhav D. R. Reddy P. M. (2006). Introgression of Cajanus platycarpus genome into cultivated pigeonpea (Cajanus cajan). Euphytica 149, 161–167. doi: 10.1007/s10681-005-9063-630311153

[B56] Mallikarjuna N. Moss J. P. (1995). Production of hybrids between C. platycarpus and C. cajan. Euphytica 83, 43–46. doi: 10.1007/BF01677859

[B57] Mallikarjuna N. Saxena K. B. (2002). Production of hybrids between Cajanus acutifolius and C. cajan. Euphytica 124, 107–110. doi: 10.1023/a:101566261287041886696

[B58] Mallikarjuna N. Senapathy S. Jadhav D. R. Saxena K. B. Sharma H. C. Upadhyaya H. D. . (2011). Progress in the utilization of Cajanus platycarpus (Benth.) Maesen in pigeonpea improvement. Plant Breed. 130, 507–514. doi: 10.1111/j.1439-0523.2011.01870.x40046247

[B59] Manik S. M. N. Pengilley G. Dean G. Field B. G. Shabala S. Zhou M. (2019). Soil and crop management practices to minimize the impact of waterlogging on crop productivity. Front. Plant Sci. 10, 140. doi: 10.3389/fpls.2019.00140 30809241 PMC6379354

[B60] Mittler R. (2002). Oxidative stress, antioxidants and stress tolerance. Trends Plant Sci. 7, 405–410. doi: 10.1016/S1360-1385(02)02312-9 12234732

[B61] Msehli S. E. Rima N. Sghaier H. Aschi-Smiti S. Brouquisse R. (2016). Impact of hypoxia on nodulation and growth of the legume plant Medicago truncatula in symbiosis with Sinorhizobium meliloti. Int. J. Curr. Res. Biosci. Plant Biol. 3, 53–60. doi: 10.20546/ijcrbp.2016.311.009

[B62] Nuruzzaman M. Manimekalai R. Sharoni A. M. Satoh K. Kondoh H. Ooka H. . (2010). Genome-wide analysis of NAC transcription factor family in rice. Gene 485, 30–44. doi: 10.1016/j.gene.2010.06.008 20600702

[B63] Ohri D. Singh S. P. (2002). Karyotypic and genome size variation in Cajanus cajan (L.) Millsp. (pigeonpea) and some wild relatives. Genet. Resour. Crop Evol. 49, 1–10. doi: 10.1023/A:101386661141041886696

[B64] Pan J. Sharif R. Xu X. Chen X. (2021). Mechanisms of waterlogging tolerance in plants: Research progress and prospects. Front. Plant Sci. 11, 627331. doi: 10.3389/fpls.2020.627331 33643336 PMC7902513

[B65] Pazhamala L. Saxena R. K. Singh V. K. Sameerkumar C. V. Kumar V. Sinha P. . (2015). Genomics assisted breeding for boosting crop improvement in pigeonpea. Front. Plant Sci. 6, 50. doi: 10.3389/fpls.2015.00050 25741349 PMC4330709

[B66] Posso D. A. da-Silva C. J. Shimoia E. P. Martins T. S. Reissig G. N. Oliveira A. C. B. . (2023). Root-hypoxia tolerance in soybean sister-lines plants indicates a better balance in energy use/dissipation and oxidative stress control. Plant Stress 10, 100225. doi: 10.1016/j.stress.2023.10022542574925

[B67] Pucciariello C. Boscari A. Tagliani A. Brouquisse R. Perata P. (2019). Exploring legume-rhizobia symbiotic models for waterlogging tolerance. Front. Plant Sci. 10, 578. doi: 10.3389/fpls.2019.00578 31156662 PMC6530402

[B68] Quimio C. A. Torrizo L. B. Setter T. L. Ellis M. Grover A. Abrigo E. M. . (2000). Enhancement of submergence tolerance in transgenic rice overproducing pyruvate decarboxylase. J. Plant Physiol. 156, 516–521. doi: 10.1016/s0176-1617(00)80167-4

[B69] Rao N. K. K. Reddy L. J. Bramel P. J. (2003). Potential of wild species for genetic enhancement of some semi-arid food crops. Genet. Resour. Crop Evol. 50, 707–721. doi: 10.1023/A:102505501895441886696

[B70] Rathinam M. Mishra P. Vasudevan M. Budhwar R. Mahato A. K. Prabha A. L. . (2019). Comparative transcriptome analysis of pigeonpea, Cajanus cajan (L.) and one of its wild relatives Cajanus platycarpus (Benth.) Maesen. PloS One 14, e0218731. doi: 10.1371/journal.pone.0218731 31269083 PMC6609033

[B71] Rocha M. Licausi F. Araújo W. L. Nunes-Nesi A. Sodek L. Fernie A. R. . (2010). Glycolysis and the tricarboxylic acid cycle are linked by alanine aminotransferase during hypoxia induced by waterlogging of Lotus japonicus. Plant Physiol. 152, 1501–1513. doi: 10.1104/pp.109.150045 20089769 PMC2832266

[B72] Safavi-Rizi V. Herde M. Stöhr C. (2020). RNA-Seq reveals novel genes and pathways associated with hypoxia duration and tolerance in tomato root. Sci. Rep. 10, 1692. doi: 10.1038/s41598-020-57884-0 32015352 PMC6997459

[B73] Sairam R. K. Kumutha D. Ezhilmathi K. Chinnusamy V. Meena R. C. (2009). Waterlogging induced oxidative stress and antioxidant enzyme activities in pigeonpea. Biol. Plant 53, 493–504. doi: 10.1007/s10535-009-0090-330311153

[B74] Sairam R. K. Kumutha D. Ezhilmathi K. Deshmukh P. S. Srivastava G. C. (2008). Physiology and biochemistry of waterlogging tolerance in plants. Biol. Plant 52, 401–412. doi: 10.1007/s10535-008-0084-630311153

[B75] Sarode S. B. Wanjari S. S. Mate S. N. (2007). Lenticel development and its role in waterlogging tolerance of pigeonpea. Ann. Plant Physiol. 21, 218–220.

[B76] Sasidharan R. Voesenek L. A. C. J. (2015). Ethylene-mediated acclimations to flooding stress. Plant Physiol. 169, 3–12. doi: 10.1104/pp.15.00387 25897003 PMC4577390

[B1313] Saxena K. B. Kumar R. V. (2010). Insect-aided natural out-crossing in four wild relatives of pigeonpea. Euphytica 173 (3), 329–335. doi: 10.1007/s10681-009-0088-0

[B78] Saxena K. B. Kumar R. V. (2003). Development of a cytoplasmic-nuclear male-sterility system in pigeonpea using C. scarabaeoides (L.) Thouars. Indian J. Genet. 63, 225–229.

[B79] Saxena K. B. Choudhary A. K. Saxena R. K. Chauhan Y. S. (2020). Can pigeonpea hybrids negotiate stresses better than inbred cultivars? Breed. Sci. 70, 423–429. doi: 10.1270/jsbbs.20015 32968344 PMC7495196

[B77] Saxena K. B. Singh L. Kumar R. V. Rao A. N. (1996). “ Development of CMS system in pigeonpea at ICRISAT asia center”, in: Proceedings of the Working Group on Cytoplasmic-Genic Male-Sterility (CMS) in Pigeonpea. (Patancheru, Hyderabad, A.P: ICRISAT), 32–50.

[B80] Sharma S. Sharma J. Soni V. Kalaji H. M. Elsheery N. I. (2021). Waterlogging tolerance: A review on regulative morpho-physiological homeostasis of crop plants. J. Water Land Dev. 49, 16–28. doi: 10.24425/jwld.2021.137092

[B81] Sharmin R. A. Karikari B. Chang F. Al Amin G. M. Bhuiyan M. R. Hina A. . (2021). Genome-wide association study uncovers major genetic loci associated with seed flooding tolerance in soybean. BMC Plant Biol. 21, 497. doi: 10.1186/s12870-021-03268-z 34715792 PMC8555181

[B83] Singh N. K. Gupta D. K. Jayaswal P. K. Mahato A. K. Dutta S. Singh S. . (2012). The first draft of the pigeonpea genome sequence. J. Plant Biochem. Biotechnol. 21, 98–112. doi: 10.1007/s13562-011-0088-8 24431589 PMC3886394

[B84] Singh S. Kudapa H. Garg V. Varshney R. K. (2021). Comprehensive analysis and identification of drought-responsive candidate NAC genes in three semi-arid tropics (SAT) legume crops. BMC Genomics 22, 289. doi: 10.1186/s12864-021-07602-5 33882825 PMC8059324

[B82] Singh A. K. Upadhyaya A. Choudhary A. K. Saurabh K. Jeet P. Monoburallah . (2022). Development of waterlogging tolerant lines of pigeonpea for Eastern India. J. AgriSearch 9, 201–207. doi: 10.21921/jas.v9i03.11002

[B86] Sultana R. (2010). Can a drowning pigeonpea perform. SA Trends 102. doi: 10.1016/b978-0-12-821450-3.00013-542574925

[B85] Sultana R. Vales M. I. Saxena K. B. Rathore A. Rao S. K. Mula M. G. . (2013). Waterlogging tolerance in pigeonpea [Cajanus cajan (L.) Millsp.]: Genotypic variability and identification of tolerant genotypes. J. Agric. Sci. 151, 659–671. doi: 10.1017/S002185961200075541292463

[B87] Suzuki N. Koussevitzky S. Mittler R. Miller G. (2012). ROS and redox signalling in the response of plants to abiotic stress. Plant Cell Environ. 35, 259–270. doi: 10.1111/j.1365-3040.2011.02336.x 21486305

[B88] Swathi D. Sabumon P. C. Maliyekkal S. M. (2017). Microbial mediated anoxic nitrification-denitrification in the presence of nanoscale oxides of manganese. Int. Biodeterioration Biodegradation 119, 499–510. doi: 10.1016/j.ibiod.2016.10.04342574925

[B89] Takele A. McDavid C. (1995). The response of pigeonpea cultivars to short durations of waterlogging. Afr. Crop Sci. J. 3, 51–58. doi: 10.4314/acsj.v3i1.54565

[B90] Tiwari T. N. Dutta D. Tewari K. Laxmi V. (2024). Physiological characterization of pigeonpea genotypes [Cajanus cajan (L.) Mill sp.] for waterlogging tolerance. Legume Res. 49, 397–405. doi: 10.18805/lr-5342

[B91] Tyagi A. Sharma S. Srivastava H. Singh P. K. Kaila T. Ali S. . (2023). Transcriptome profiling of two contrasting pigeonpea (Cajanus cajan) genotypes in response to waterlogging stress. Front. Genet. 13, 1048476. doi: 10.3389/fgene.2022.1048476 36704340 PMC9871235

[B92] Upadhyaya H. D. Sharma S. Reddy K. N. Saxena R. K. Varshney R. K. Gowda C. L. L. (2013). “ Pigeonpea,” in Genetic and Genomic Resources of Grain Legume Improvement (Amsterdam: Elsevier), 181–202. doi: 10.1016/B978-0-12-397935-3.00008-6

[B93] Varshney R. K. Bohra A. Yu J. Graner A. Zhang Q. Sorrells M. E. (2021). Designing future crops: Genomics-assisted breeding comes of age. Trends Plant Sci. 26, 631–649. doi: 10.1016/j.tplants.2021.03.010 33893045

[B94] Varshney R. K. Chen W. Li Y. Bharti A. K. Saxena R. K. Schlueter J. A. . (2011). Draft genome sequence of pigeonpea (Cajanus cajan), an orphan legume crop of resource-poor farmers. Nat. Biotechnol. 30, 83–89. doi: 10.1038/nbt.2022 22057054

[B95] Watson A. Ghosh S. Williams M. J. Cuddy W. S. Simmonds J. Rey M. D. . (2018). Speed breeding is a powerful tool to accelerate crop research and breeding. Nat. Plants 4, 23–29. doi: 10.1038/s41477-017-0083-8 29292376

[B96] Weits D. A. Giuntoli B. Kosmacz M. Parlanti S. Hubberten H. M. M. Riegler H. . (2014). Plant cysteine oxidases control the oxygen-dependent branch of the N-end-rule pathway. Nat. Commun. 5, 3425. doi: 10.1038/ncomms4425 24599061 PMC3959200

[B97] Yamauchi T. Abe F. Tsutsumi N. Nakazono M. (2019). Root cortex provides a venue for gas-space formation and is essential for plant adaptation to waterlogging. Front. Plant Sci. 10, 259. doi: 10.3389/fpls.2019.00259 31024577 PMC6465681

[B98] Yamauchi T. Nakazono M. (2022). Mechanisms of lysigenous aerenchyma formation under abiotic stress. Trends Plant Sci. 27, 13–15. doi: 10.1016/j.tplants.2021.10.001 34810105

[B99] Yamauchi T. Yoshioka M. Fukazawa A. Mori H. Nishizawa N. K. Tsutsumi N. . (2017). An NADPH oxidase RBOH functions in rice roots during lysigenous aerenchyma formation under oxygen-deficient conditions. Plant Cell 29, 775–790. doi: 10.1105/tpc.16.00976 28351990 PMC5435434

[B100] Yang J. Zhang S. Li H. Wang L. Liu Y. Niu L. . (2021). Genome-wide analysis and characterization of R2R3-MYB family in pigeonpea (Cajanus cajan) and their functional identification in phenylpropanoid biosynthesis. Planta 254, 64. doi: 10.1007/s00425-021-03713-1 34487243

[B101] Yu W. W. Chen Q. F. Liao K. Zhou D. M. Yang Y. C. He M. . (2024). The calcium-dependent protein kinase CPK16 regulates hypoxia-induced ROS production by phosphorylating the NADPH oxidase RBOHD in Arabidopsis. Plant Cell 36, 3451–3466. doi: 10.1093/plcell/koae153 38833610 PMC11371159

[B102] Zaman M. S. U. Malik A. I. Luna L. N. Hossain M. A. Alam A. K. M. M. Prodhan M. A. . (2024). Differences of waterlogging tolerance in winter pulse crop between emergence and vegetative stages. J. Agron. Crop Sci. 210, e12704. doi: 10.1111/jac.1270440046247

[B103] Zhang J. McDonald S. C. Wu C. Ingwers M. W. Abdel-Haleem H. Chen P. . (2022). Quantitative trait loci underlying flooding tolerance in soybean (Glycine max). Plant Breed. 141, 236–245. doi: 10.1111/pbr.1300840046247

[B104] Zubrycka A. Dambire C. Carbonare L. D. Sharma G. Boeckx T. Swarup K. . (2023). ERFVII action and modulation through oxygen-sensing in Arabidopsis thaliana. Nat. Commun. 14, 4665. doi: 10.1038/s41467-023-40366-y 37537157 PMC10400637

